# Techno-Economic Analysis of the Production of Liquid Biofuels from Sewage Sludge via Hydrothermal Liquefaction

**DOI:** 10.1021/acs.energyfuels.2c03647

**Published:** 2022-12-27

**Authors:** Gonzalo Del Alamo, Mette Bugge, Thomas Helmer Pedersen, Lasse Rosendahl

**Affiliations:** † Thermal Energy Department at SINTEF Energy Research, Trondheim7491, Norway; ‡ Department of Energy, Aalborg University, Aalborg9100, Denmark

## Abstract

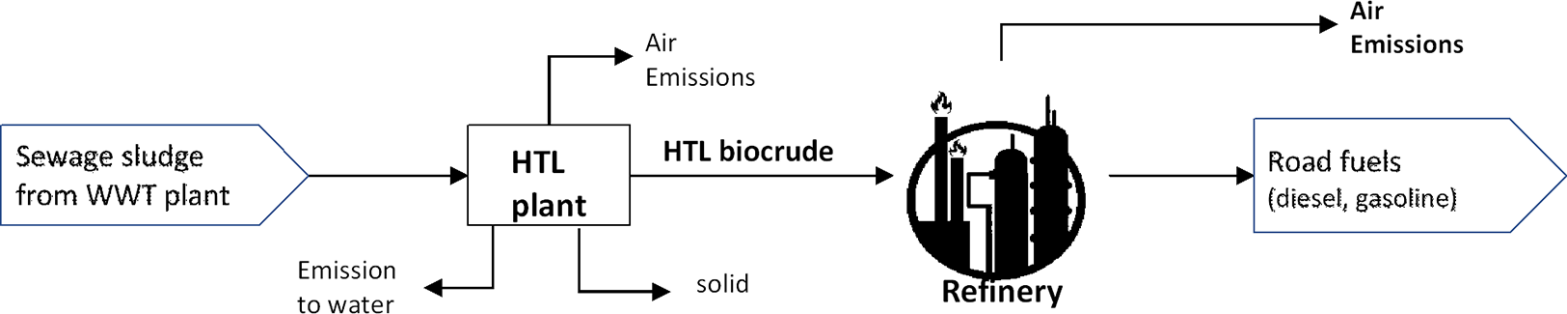

This work addresses the process and economic performance of the production of gasoline and diesel range fuels from urban sewage sludge. The overall production route involves direct conversion of the sewage sludge to an intermediate oil phase, so-called biocrude, via hydrothermal liquefaction at near-critical water conditions and further upgrading of the biocrude based on conventional refinery processes. The overall mass and energy yields of combined naphtha and middle distillate from sewage sludge on dry basis are approximately 19 and 60%, where the naphtha fraction represents about 45% of the total, with a minimum fuel selling price ranging between 2.4 and 0.8 €/liter assuming full investment in both the biocrude production and upgrading plant with sewage sludge feed capacities in the range of 3 to 30 dry-ton/day. If existing equipment at refinery can be used for upgrading of the biocrude, the minimum fuel selling price can be reduced by approximately 7%.

## 1 Introduction

The use of advanced biofuels has been included as part of a wider global strategy for reducing GHG emissions in the transport sector.^1^ The role of advance biofuels is particularly relevant for achieving the decarbonization targets in the heavy-duty transport sectors, i.e., marine, aviation, and long-haul road transport, where the use of other alternative renewable energy carriers like electricity or hydrogen is not feasible. The total demand of liquid biofuels worldwide is estimated to be approximately 27% of the total fuel needed by 2050, with a similar share of about 25% to be reached within the European Union by 2030. However, despite the high short-term market demand, progress in commercialization of advanced liquid biofuels is still limited. One main reason is the high capital and operating costs of these technologies^2^ that require large scales to reach reasonable production costs. The need of larger plant capacities significantly increases the cost of feedstock transport^3^ as well as the risk of assuring a continuous supply of the feedstock throughout the lifetime of the plant. These reasons lead to financial risks when evaluating the viability of commercial projects, which hinder the commercial realization.

This work addresses the economic feasibility of producing liquid biofuels from urban sewage sludge. The overall conversion route considered the decentralized conversion of sewage sludge to biocrude via hydrothermal liquefaction (HTL) at near-critical-water conditions and further upgrading of the biocrude to liquid biofuels based on conventional refinery processes. Therefore, the analysis explores two important strategies for improving the overall economy of producing advance liquid biofuels, i.e., to lower the feedstock supply cost by utilizing low-grade organic waste and to reduce capital cost by utilizing of existing petrochemical infrastructures for refining of organic intermediates to marketable biofuels. Sewage sludge has been considered in this analysis as a model feedstock representing renewable urban waste. Sewage sludge is an abundant feedstock, with an annual production in the European Union being approximately 10 million tons^4^ in 2019. At present, the commercial disposal of sewage sludge includes the direct spreading in soils,^5,6^ anaerobic digestion for production of biogas,^7^ thermal conversion for production of heat and power,^8−10^ and co-combustion in kilns for cement production.^11^ Due to its high moisture content, typically above 80% wt., the transport and direct combustion of sewage sludge is energetically unfavorable. It also causes severe fouling problems in boilers due to alkali and phosphorous contents.^12^ Anaerobic digestion of sewage sludge achieves high methane yields in the range of 230–430 Nm^3^ CH_4_/ton volatile solid^13^ depending on the pretreatment methods and digestion conditions. However, it requires typically large conversion times leading to high reactor volumes and thus high capital investment.^10^ In this context, hydrothermal liquefaction (HTL) technology exhibits several advantages for the conversion of wet organic waste fractions to energy.^14−16^ Under near critical or near-critical conditions, liquid water exhibits high ionic product.^17,18^ These conditions enhance the decomposition of macromolecular constituents of the feedstock by ionic reactions taking place in the liquid water with formation of a high-energy density oil phase,^17^ or so-called biocrude. Depending on the operational conditions and feedstock characteristics, conversion times in the HTL process are typically below 30 min,^15^ which is about three orders of magnitude lower than the conversion to biogas in anaerobic digestion. Moreover, the chemical and physical characteristics of the biocrude produced from HTL make it compatible with fossil-based crude oil for upgrading to liquid fuels in conventional refinery processes.^19^

The techno-economic analysis of the production of liquid biofuels from sewage sludge based on HTL technology has been addressed in the literature.^19,20^ These two studies by the same author considered decentralized production of biocrude with centralized upgrading at refinery. The former study assumed the same design and product yields for the HTL process as considered earlier^21^ for the conversion of algae showing a minimum biocrude selling price (MBSP) of $_2015_ 31.6/GJ and a minimum fuel selling price (MFSP) of $_2015_ 40.75/GJ for a base conversion capacity of 100 dry ton/day covered by 10 identical HTL plants for production of biocrude. The later study by the same author performs a more detailed evaluation of the HTL and upgrading processes for several wet waste fractions based on measurements of the product yields and composition from engineering-scale and bench-scale experiments. The results from this work on the conversion of sewage sludge generated at waste-water treatment (WWT) plants showed values of the MBSP per unit volume and energy of 0.67 $_2021_/liter and 19.5 $_2021_/GJ, respectively, and the MFSP per unit volume diesel equivalent and per unit total hydrocarbons products energy of 1.02 $_2021_/liter and 29.8 $_2021_/GJ, respectively. This analysis assumed a decentralized HTL plant with a constant capacity of 110 dry ton/day and centralized upgrading at refinery also with a constant capacity of 30.4 m^3^/day. The process design of the HTL plant considered treatment of the HTL process water by aqueous catalytic upgrading followed by ammonia stripping before disposal back to the WWT plant. Other investigation addressing the techno-economics of hydrothermal liquefaction for the conversion of wet organic waste of biocrude^22^ has shown MBSP values in the range of 22 and 41 $_2020_/GJ. The analysis presented in this paper contributes to the topic of techno-economics of HTL biofuel production from sewage sludge in several relevant aspects. It provides a detailed description of the process design that includes the main process and auxiliary equipment, allowing a realistic estimation of the capital cost. The analysis has been performed through parametric models for evaluating the main material and energy flows from experimental results as well as for evaluating the main size of equipment. This parametric approach has been used for scaling up the process and evaluating the economy as a function of the production capacity.

## 2 Description of the Process Design

### 2.1 Production of Biocrude from Sewage Sludge

**Figure 1:**
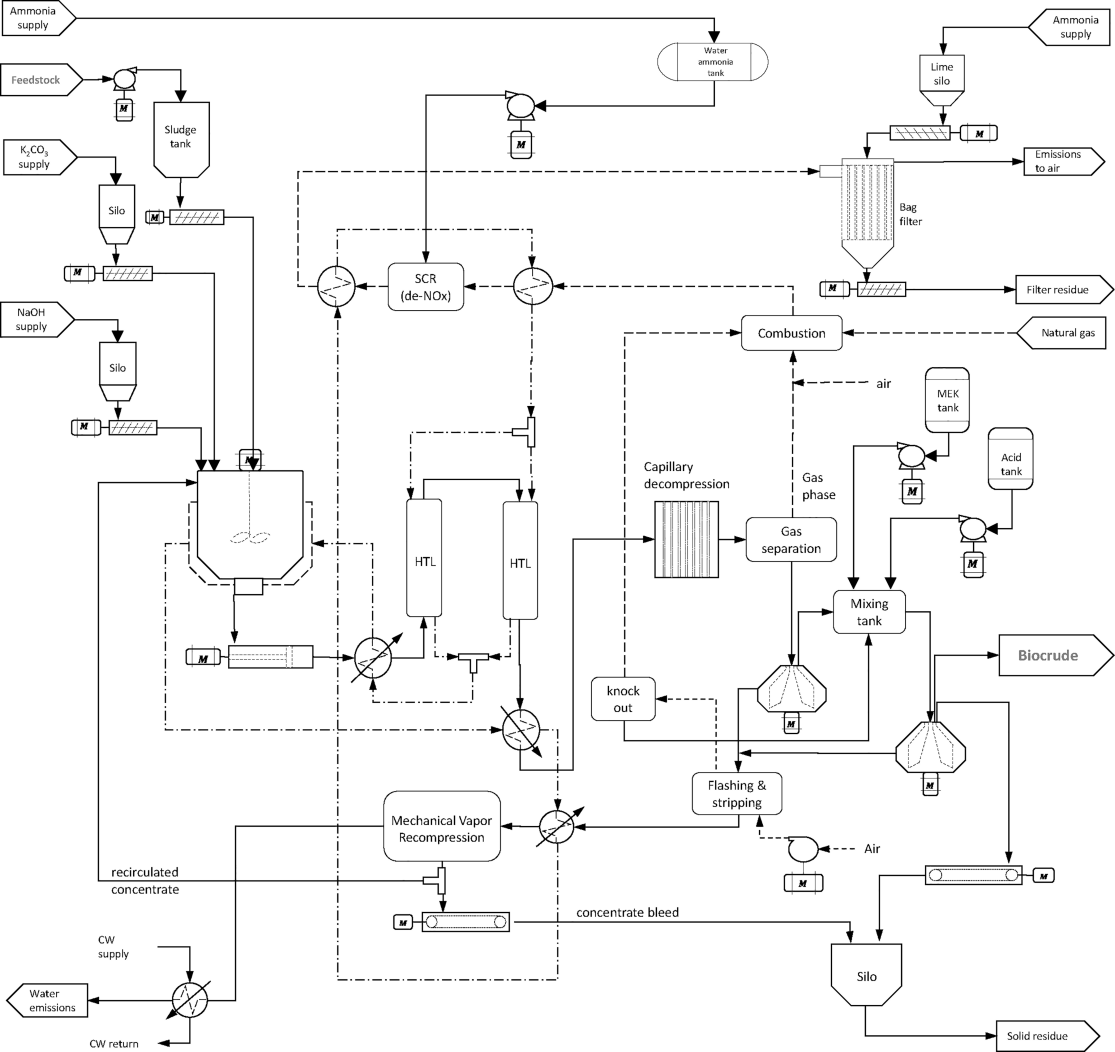
Schematic representation of the biocrude production process from sewage sludge by hydrothermal liquefaction.

The process design for the overall conversion of sewage sludge to biocrude is shown in Figure 1. The raw feedstock is stored in a buffer tank and transported by a screw conveyor into the slurry preparation tank where it is mixed with the catalyst (K_2_CO_3_), the base (NaOH), and a fraction of the concentrate from the HTL water treatment. The slurry preparation tank is designed as a stirred tank to ensure uniform mixing of the slurry with a heating jacket to increase the temperature of the slurry for reducing the viscosity and thus improving the pumpability. The slurry is discharged from the stirred tank by a pump that feeds the main pressurization pump, which increases the pressure of the slurry to the HTL conditions. The main pressurization pump is designed as a single piston low stroke reciprocating pump. The pressurized slurry is heated to the HTL conditions in a U-tube heat exchanger and then fed into the HTL reactor. The process design considers one or several parallel HTL reactors depending on the capacity of the biocrude production plant. The HTL reactor is equipped with a heating jacket and dimensioned to achieve the required residence time to complete the liquefaction of the slurry. The HTL product is a multiphase flow consisting of a binary liquid mixture of aqueous and oil phases with dispersed non-dissolved solids and a gas phase. The product from the HTL reactor is directly cooled down in a heat exchanger before depressurization. Heating of the slurry and cooling of the HTL product are performed in a closed loop using a thermal fluid that recovers the heat from cooling of the HTL product. The cooled liquefaction product is partially depressurized in capillary columns to an intermediate pressure of about 30 bar-g and then taken to a gas separator where the gas phase is separated from the oil, aqueous, and solid phases. These three phases leave the separator as an oil–sand emulsion and stored in a buffer tank for further separation in a two-stage centrifugation system. The first centrifuge separates a fraction of the water. The remaining oil sand emulsion from the first centrifuge is mixed with acid, methyl ethyl ketone (MEK), and a condensed light oil stream for breaking the oil/water chemical binding and then fed into the second centrifuge where the oil, solid, and aqueous phases are separated. MEK is here used only during startups of the plant since the condensed light oil effectively can be effectively used as a solvent to break up the oil–sand emulsion. The solids from the second centrifuge are collected in a hopper and transported by a screw conveyor to a silo. The oil from the centrifuge, here also called biocrude, is stored in a tank for further delivery. The aqueous phase from the second centrifuge is discharged into a flash tank. Compressed air is injected into the flash tank for stripping of the ammonia, light oils, and the MEK solvent. The gas stream from the flash tank is further cooled for condensation of the light oils and MEK, which are recirculated and mixed with the oil–sand emulsion before the second centrifuge, and the remaining gas stream is used as a combustion air in the HTL gas treatment. The HTL process water from the flash tank is stored in a buffer tank before treatment.

Treatment of the process water is achieved by partial evaporation of the process water in a mechanical vapor recompression (MVR) unit. The MVR technology has been selected in this analysis since it is commercially available and has been successfully used by Steeper Energy at a demo scale for treating HTL water derived from woody biomass and at a pilot scale for treating HTL water derived from sewage sludge. The MVR system effectively utilizes the latent heat from the vapor for partial evaporation of the inlet process water. The concentrate from the MVR unit is partially recirculated back to the slurry preparation tank before the HTL process, and the remaining concentrate bleed is transported by a screw conveyor to the solid residue silo. The cleaned condensate from the MVR unit is further cooled and stored in a buffer tank before disposal. The gas separated from the HTL product is directly combusted in a burner to produce the heat required by the process. The calorific value and flammability of the HTL gas product are typically low due to the high CO_2_ concentration. Therefore, the HTL gas is co-combusted with natural gas to provide additional thermal power to cover the total heat demand by the plant and compensate the variability in the energy content in the HTL gas. The flue gas from the combustion process contains SO_2_ and NOx and requires further cleaning to fulfill the air emission regulations. The first step in the flue gas cleaning is a selective catalytic reduction (SCR) of the NOx where urea is used as a reducing agent. After NOx reduction, flue gas is further cooled down and taken into a dry scrubber for removal of acid gases SO_2_ with hydrated lime. The scrubber is equipped with bag filters to remove the reacted hydrated lime, which is removed from the external surface of the bag filters by pulsing compressed air, collected at the bottom of the scrubber, and transported by a screw conveyor into a silo. The heat from the flue gas is recovered to the thermal oil by two heat exchangers before the SCR unit and the scrubber. The main process design parameters for the biocrude production process are listed in Table 1.

**Table 1:** Process Parameters for the Biocrude Production Process Considered in the Analysis

process parameter	value
storage capacity sewage sludge (h)	12
slurry preparation temperature (deg. C)^a^	120
slurry preparation pressure (bar-a)	5
agitation at slurry preparation tank (rpm)	150
slurry dry matter concentration (% wt.)	25
slurry pH	9
base consumption in HTL (% wt. input wet feedstock)	0.34 (NaOH)
catalyst consumption in HTL (% wt. input wet feedstock)	0.14 (K_2_CO_3_)
HTL temperature (deg. C)	350
HTL pressure (bar-g)	320
HTL residence time (s)	1500
HTL heating rate (deg. C/s)	2.0
gravimetric separation temperature (deg. C)	150
gravimetric separation pressure (bar-g)	30
temperature at aqueous effluent flash tank (deg. C)	100
pressure at aqueous effluent flash tank (bar-a)	1.2
temperature at first centrifugation	150
aqueous/oil/ash content after first centrifugation	0.5/2/1
centrifuge rotational speed (rpm)	9510
citric acid consumption oil separation (mol/liter emulsion)	0.013 [1]
MEK consumption oil separation (kg/kg oil)^a^	1.0 [1]
MEK recovery (%)^a^	98
MVR operating temperature (deg. C)	110
MVR operating pressure (bar-a)	1.013
MVR concentration factor (% wt. water reduction)	80
recirculation of MVR concentrate to HTL process (%)	80
boiler pressure (bar-g)	–0.05
excess air in combustion	1.1
combustion air supply pressure (kPa-g)	1.3
natural gas supply pressure (kPa-g)	1.3
SCR temperature (deg. C)	390
ammonia consumption SCR (kg/Nm^3^)	0.43
SCR, electricity consumption (kWh/Nm^3^)	1.4 × 10^–3^
SCR, catalyst lifetime (hours)	40,000
dry scrubber temperature (deg. C)	140
lime consumption at dry scrubber (g/Nm^3^)	2
thermal fluid	thermal oil
thermal fluid supply temperature (deg. C)	400
thermal fluid supply pressure (bar-g)	15
dry matter content in the leaching tank	10%
operating temperature at HTL solids leaching tank (deg. C)	95
operating pressure at HTL solids leaching tank (bar-a)	1.013
acid concentration at HTL solids leaching tank	0.4 M (H_2_SO_4_)

a During plant start.

### 2.2 Biocrude Upgrading to Naphtha and Middle Distillate

**Figure 2:**
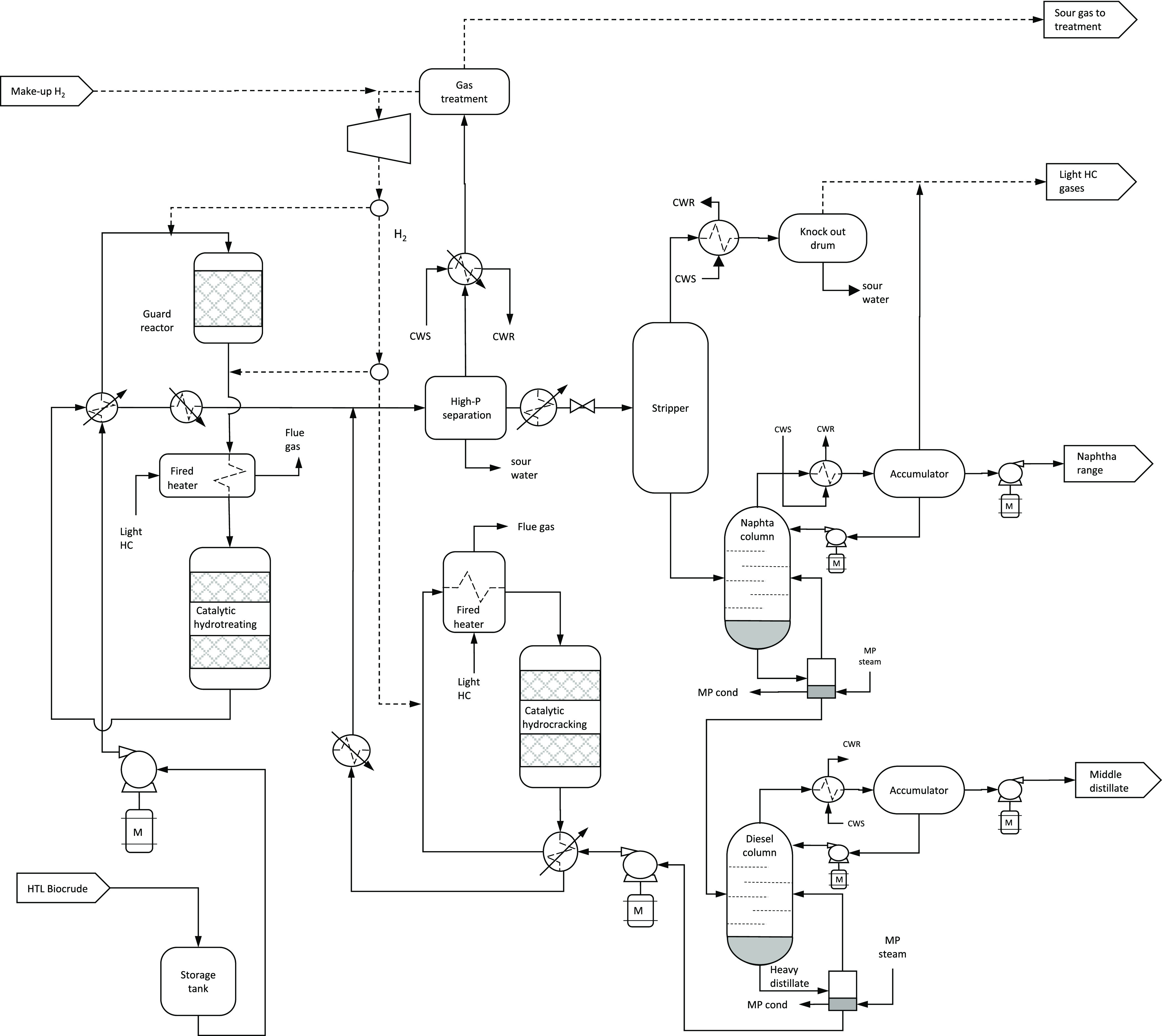
Schematic representation of the biocrude upgrading process.

The process design of the biocrude upgrading is graphically represented in Figure 2. The raw biocrude is stored in a buffer tank before treatment. From the buffer tank, the biocrude is pumped, mixed with hydrogen, and heated before entering the guard reactor, where inorganic heteroatoms in the biocrude are reduced and adsorbed by the catalyst. Heating of the feed is performed in a heat exchanger with recovery of thermal energy from the hydrotreating product. The product from the guard reactor is mixed with hydrogen, heated, and taken into the hydrotreating reactor where the N, S, and O heteroatoms are reduced and separated from the oil. Heating of the feed before hydrotreating is performed in a fired heater using light hydrocarbons separated during fractionation of the hydrotreated oil as a gas fuel. The product from the hydrotreating reactor is cooled by and taken to a high-pressure (HP) three-phase gravimetric separator where a light sour gas and water are separated from the organic liquid phase. The organic liquid from the HP separator is heated and enters a second low-pressure (LP) stripper where light hydrocarbons and water vapor are separated and condensed. The process water streams from the HP and LP separation are disposed to the water treatment system at the refinery. The oil phase from the LP stripper is fed directly into a first distillation column for separation of naphtha, with boiling point in the range of 90–210 deg. C. The bottom distillate from the naphtha column is re-boiled and fed into a second distillation column for separation of middle distillate, with boiling point in the range of 210 and 310 deg. C. The heavy distillate residue from the diesel column is pumped, mixed with hydrogen, heated, and fed into a hydrocracking reactor where the heavy distillate is converted to naphtha and middle distillate, and the remaining S, N, and O contained in the feed are reduced and separated from the liquid organic product. The product from the hydrocracking is cooled and mixed with the hydrotreating product before phase separation. The light sour gas from the HP separation, containing non-reacted H_2_, CO_2_, NH_3_, H_2_S, and light hydrocarbons, is further treated in an absorption column where rich amine is injected for solubilization of CO_2_, NH_3_, and H_2_S. The H_2_ enriched gas stream from the amine column is mixed with make-up hydrogen, compressed, and recirculated back to the guard, hydrotreating, and hydrocracking reactors. The lean amine with dissolved gases is heated and pumped into a stripper where the gases are desorbed from the amine. The rich amine after the stripper is recirculated back to the absorption column prior cooling. The off gas from the stripper is disposed to the refinery for further treatment. Table 2 lists the main process design parameters used for the upgrading of the HTL biocrude to naphtha and middle distillate.

**Table 2:** Process Parameters for the Overall Biocrude Upgrading Process Considered in the Analysis

process parameter	value
storage capacity biocrude (h)	12.00
preheated biocrude temperature before pumping (deg. C)	40.00
guard reactor temperature	290.00
guard reactor pressure	100.00
guard reactor hydrogen consumption (Nm^3^/m^3^ feed)	574
guard reactor hydrogen reacted (% wt. feed)	0.52
guard reactor catalyst WHSV (kg/kg/h)	0.40
guard reactor catalyst lifetime (h)	16000.00
hydrotreating temperature	360.00
hydrotreating pressure	100.00
hydrotreating hydrogen consumption (Nm^3^/m^3^ feed)	1747
hydrotreating hydrogen reacted (% wt. feed)	1.57
hydrotreating catalyst WHSV (kg/kg/h)	0.40
hydrotreating catalyst lifetime (h)	16000.00
hydrocracking temperature (deg. C)	370.00
hydrocracking pressure (bar-g)	100.00
hydrocracking hydrogen consumption (Nm^3^/m^3^)	2400.00
hydrocracking hydrogen reacted (% wt. feed)	5.4
hydrocracking catalyst WHSV (kg/kg/h)	0.50
hydrocracking catalyst lifetime (h)	16000.00
temperature at three-phase high-pressure separator (deg. C)	40.00
pressure at three-phase high-pressure separator	100.00
temperature at three-phase low-pressure separator (deg. C)	240.00
pressure at three-phase low-pressure separator	2.00
inlet temperature at distillation column (deg. C)	400.00
pressure distillation column (bar-g)	1.013
naphtha cut-off temperature at distillation (deg. C)	95
diesel cut-off temperature at distillation (deg. C)	210
heavy-fraction cut-off boiling point (deg. C)	310.00
diesel range cut-off boiling point (deg. C)	210.00
naphtha cut-off boiling point (deg. C)	80.00
amine absorber temperature (deg. C)	40.00
amine absorber pressure (bar-g)	30.00
make-up amine consumption (% wt. feed gas)	7.00
heating amine plant (MJ/kg feed gas)	0.272
net cooling amine plant (MJ/kg feed gas)	0.40
electric power consumption amine plant (kWh/kg feed gas)	46.50

## 3 Process Models and Analysis

### 3.1 Flow Properties

The process analysis considers a general multi-phase slurry flow structure, with physical properties evaluated as a function of the composition and properties of the phases. For flows with solid particle dispersion, it has been assumed that the particles are suspended and distributed uniformly in the liquid phase. Then, the effects of particle interaction on the molecular transport properties can be neglected. The apparent density and the specific heat and enthalpy of the flow are calculated, respectively, from ρ = ∑ ϕ_*k*_ρ_*k*_, *c_p_* = ∑ *y_k_c*_*p*, *k*_, and *h* = ∑ *Y_k_h_k_*, where ϕ_*k*_, *y_k_*, ρ_*k*_, *c*_*p*, *k*_, and *h_k_* denote the void fraction, mass fraction, density, specific heat, and specific enthalpy of each phase. The effective viscosity of the slurry^23^ is evaluated from μ̅ = μ_*l*_μ_*r*_, where μ_*l*_ represents the viscosity of the liquid phase and μ_*r*_ denotes a relative viscosity dependent on the particle geometry and concentration. The relative viscosity of the slurry feed prepared from sewage sludge is calculated as a function of the total solids volume fraction using the correlation μ_*r*_ = 1 + ϕ*_s_A*/(1 – ϕ_*s*_/*B*), with the constants *A* = 3000 and *B* = 0.27 estimated from measurements of the total dynamic viscosity of sewage sludge slurries at 320 bar-g. The thermal conductivity is calculated as a function of the thermal conductivity of the solid *k_s_* and liquid *k_l_* phases from *k̅* = *k_l_k_r_*, where *k_r_* is the relative thermal conductivity of the slurry evaluated from *k_r_* = 1 + 3ϕ_*s*_β + 3ϕ_*s*_^2^β^2^[1 + (β/4)(β + 29/4)/(5 + β)], with β = [(*k_s_*/*k_l_*) – 1]/[(*k_s_*/*k_l_*) + 2] denoting the solid to liquid thermal conductivity ratio. The physical properties of water have been evaluated using the IAPWS formulation.^24^ When the molecular composition is defined, evaluation of the enthalpy and specific heat has been calculated using the NIST database of thermodynamic properties of fluid systems.^25^

**Table 3:** Experimental Measurements of the Oil, Aqueous, and Solid Phases Produced from Hydrothermal Liquefaction of Sewage Sludge at 320 bar-g and 350 deg. C at the Aalborg Pilot Plant^a^

feedstock	oil phase		aqueous phase		solid phase		gas phase	
HHV	MJ/kg daf	35.2^a^	MJ/kg db.	14.4	MJ/kg db.	2.38^b^	MJ/kg db.	7.1^b^
water	wt. %	1.8^a^	wt. %	91.4^b^	wt. %	35^c^	wt. %	60^c^
carbon (total organic carbon)	wt. % daf	77.4^a^	g/L	45.3 (35.4)^a^	wt. %	17.3^b^	wt. % db.	31.6^b^
hydrogen H	wt. % daf	9.8^a^	g/L		wt. %	2.1^b^	wt. % db.	1.4^b^
oxygen O	wt. % daf	8.2^a^	g/L		wt. %	3.7^b^	wt. % db.	61.2^b^
nitrogen N (N as NH_4_^+^)	wt. % daf	3.7^a^	g/L	11.6 (7.98)^a^	wt. %	1.2^b^	wt. % db.	2.45^b^
sulfur S	wt. % daf	0.8^a^	g/L	0.648^a^	wt. %	0.48^b^	wt. % db.	3.3^b^
phosphorous P	g/kg dry	0.03^a^	g/L	0.7^a^	g/kg	78.1^b^	g/kg dry	0.0
calcium Ca	g/kg dry		mg/L	31.37^a^	g/kg	34^b^	g/kg dry	0.0
aluminum Al	g/kg dry	0.0739^a^	mg/L	65.23^a^	g/kg	13.8^b^	g/kg dry	0.0
iron Fe	g/kg dry	0.11^a^	mg/L	66.27^a^	g/kg	64.2^b^	g/kg dry	0.0
magnesium Mg	g/kg dry	1.84 × 10^–3^^a^	mg/L	8.64^a^	g/kg	10.6^b^	g/kg dry	0.0
potassium K	g/kg dry	7.03 × 10^–2^^a^	mg/L	10,054^a^	g/kg	22.5^b^	g/kg dry	0.0
carbon dioxide CO_2_							% vol. dry	75.6^a^
carbon monoxide CO							% vol. dry	0.7^a^
hydrogen H_2_							% vol. dry	4.2^a^
ammonia NH_3_							% vol. dry	8.5^b^
hydrogen sulfide H_2_S							% vol. dry	0.4^b^
total hydrocarbons							% vol. dry	10.6^b^

a Notes: ^a^Measured; ^b^Calculated; ^c^Assumed; The composition of hydrocarbons in the gas phase is assumed to be a mixture of methane, ethene, ethane, propane, methanol, ethanol, and acetone with molar distributions of, respectively, 0.42, 0.26, 0.01, 0.16, 0.05, and 0.06 based on data reported by Jensen et al.^17^ on near-critical liquefaction of lignocellulosic biomass.

### 3.2 Hydrothermal Liquefaction

The conversion of the slurry in the overall hydrothermal liquefaction system, including phase separation, has been described in terms of the mass flow rate of the slurry entering the HTL process *Ṁ*_*sl*_^*HTL*^, the yields of mass *m*_*k*_^*HTL*^and chemical energy *e*_*k*_^*HTL*^, the atomic composition *y*_*k*, *i*_^*HTL*^of the products after the phase separation, and the overall heat demand *Q*_*th*_^*HTL*^. Here, the subscripts *i* and *k* denote the atomic composition and the oil (*O*), gas (*G*), solid (*S*), and aqueous (*A*) phases. The mass flow rate of the slurry entering the HTL process is calculated from *Ṁ*_*sl*_^*HTL*^= *Ṁ*_*F*_^*HTL*^(1 + *y*_*b*_^*HTL*^+ *y*_*cat*_^*HTL*^), where *y*_*cat*_^*HTL*^and *y*_*b*_^*HTL*^denote the mass fraction of the catalyst and base required by the HTL process. The product yields and composition have been evaluated semi-empirically throughout the so-called transfer coefficients *f*_*i*, *k*_^*HTL*^and *f*_*A*, *k*_^*HTL*^defined as the distribution of the atomic mass and aqueous phase from the input slurry among the different products after phase separation. From these definitions, the mass yield and the dry atomic composition of the oil, solid, aqueous, and gas streams after phase separation are calculated, respectively, from
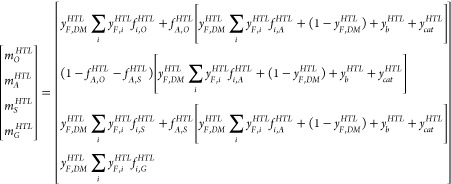
1*y*_*i*, *k*_^*HTL*^= *m*_*i*, *k*_/∑*_i_m*_*i*, *k*_ with *m*_*i*, *k*_ = *y*_*F*, *DM*_^*HTL*^*y*_*F*, *i*_^*HTL*^*f*_*k*, *i*_^*HTL*^+ *f*_*A*, *k*_^*HTL*^(*y*_*F*, *DM*_^*HTL*^*f*_*i*, *A*_^*HTL*^+ *y*_cat_^*HTL*^*y*_*i*, *cat*_ + *y*_*b*_^*HTL*^*y*_*i*, *b*_) for *k* = *O*, *S*, *G* and *m*_*i*, *A*_^*HTL*^= (1 – *f*_*A*, *O*_^*HTL*^– *f*_*A*, *S*_^*HTL*^)[*y*_*F*, *DM*_^*HTL*^∑*_i_y*_*F*, *i*_^*HTL*^*f*_*i*, *A*_^*HTL*^+ (1 – *y*_*F*, *DM*_^*HTL*^) + *y*_*b*_^*HTL*^+ *y*_*cat*_^*HTL*^]. The energy yield has been evaluated from *e*_*k*_^*HTL*^ = (*HHV*_*k*_^*HTL*^∑*_i_y*_*k*, *i*_^*HTL*^)/(*y*_*F*, *DM*_^*HTL*^*HHV*_*F*_^*HTL*^), where *HHV*_*k*_^*HTL*^ are the high heating value of each product phase. In this formulation, *Ṁ*_*F*_^*HTL*^, *y*_*F*, *DM*_^*HTL*^, *y*_*F*, *i*_^*HTL*^, and *HHV*_*F*_^*HTL*^ denote the mass flow rate, the dry matter content, the dry atomic composition, and the dry high heating value of the sewage sludge. It has been assumed that both the base and the catalyst are fully dissolved in the water and remain unreacted during conversion in the liquefaction process. Also, the process water in the oil, solid, and aqueous phases from phase separation after liquefaction has been assumed to have the same chemical composition. Values of the coefficients *f*_*i*, *k*_^*HTL*^, *f*_*A*, *k*_^*HTL*^, and *e*_*k*_^*HTL*^are shown in Table 4, evaluated using the measurements of the composition and calorific value of the HTL product phases shown in Table 3. In this table, the measurements of concentrations in the aqueous phase have been already reported by Sayegh et al.^26^ It has been assumed that the nitrogen and sulfur content in the gas phase is in the form of NH_3_ and H_2_S and that the composition of hydrocarbons in the gas phase is a mixture of methane, ethene, ethane, propane, methanol, ethanol, and acetone with molar distributions of, respectively, 0.42, 0.26, 0.01, 0.16, 0.05, and 0.06 based on the data reported by Jensen et al.^17^ on near-critical liquefaction of lignocellulosic biomass. The net rate of heat demand in the overall HTL system is calculated from *Q*_*th*_^*HTL*^= *Ṁ*_*F*_^*HTL*^[(1 – *y*_*F*, *DM*_^*HTL*^)(*h*_*w*_^*PS*^– *h*_*w*_^0^) + *y*_*F*, *DM*_^*HTL*^*c*_*F*, *DM*_(*T_HTL_* – *T*_0_) – ∑*_k_m*_*G*_^*HTL*^(1 – *y*_*G*, *H*_2_*O*_^*PS*^)*c*_*p*, *k*_(*T_HTL_* – *T*_*k*_^*PS*^)], where (*h*_*w*_^*PS*^– *h*_*w*_^0^) is the relative enthalpy of water between phase separation and ambient conditions, *T_HTL_* is the operating temperature of the HTL process, and *T*_*k*_^*PS*^and *c*_*p*, *k*_ are the temperature and the specific heat capacity on dry basis of each product from phase separation. It has been assumed that specific heat capacity of the dry matter in the sewage sludge and the solid and aqueous phases is the same and equal to 1.2 kJ/kg K. The specific heat capacity of the dry gas stream has been calculated based on the molecular composition shown in Table 3.

**Table 4:** Estimated Transfer Coefficients for the for Hydrothermal Liquefaction of Sewage Sludge and Woody Biomass Performed at Near-Critical Water Conditions at the Aalborg Pilot Plant

	oil phase	aqueous phase	solid phase	gas phase
water	0.16%	77.7%	20.2%	1.9%
chemical enthalpy	73.38%	17.14%	4.57%	4.87%
total dry matter	28.4%	23.1%	38.8%	28.4%
carbon, C	60.0%	28.5%	5.0%	6.5%
hydrogen, H	44.0%	40.0%	12.0%	4.0%
oxygen, O	7.0%	25.0%	52.0%	16.0%
nitrogen, N	21.0%	62.0%	6.0%	11.0%
sulfur, S	24.0%	14.0%	7.0%	55.0%
phosphorous, P	0.5%	7.5%	92.0%	3.1%
calcium, Ca	0.0%	6.0%	94.0%	0.0%
aluminum, Al	2.5%	11.0%	86.5%	0.0%
iron, Fe	1.4%	4.0%	94.6%	0.0%
magnesium, Mg	0.1%	2.0%	97.9%	0.0%
potassium, K	2.0%	75.1%	22.9%	0.0%

### 3.3 HTL Water Treatment by Mechanical Vapor Recompression (MVR)

The HTL water treatment in the mechanical vapor recompression (MVR) unit is defined in terms of the concentration factor η^*MVR*^, defined as the ratio between the input mass flow rate of process water and the mass flow rate of concentrate, the net heat demand *Q̇*_*th*_^*MVR*^and the net electric power consumption *Ẇ*_*el*_^*MVR*^. The mass flow rates of concentrate and cleaned water are then calculated from *Ṁ*_*c*_^*MVR*^= *Ṁ*_*A*_^*HTL*^/η^*MVR*^ and *Ṁ*_*w*_^*WT*^= *Ṁ*_*A*_^*HTL*^(1 – η^*MVR*^)/η^*MVR*^. The net heat demand is calculated from *Q̇*_*th*_^*MVR*^= *Ṁ*_*F*_^*HTL*^*m*_*A*_^*HTL*^[*y*_*A*, *H*_2_*O*_^*PS*^(*h*_*w*_^*MVR*^– *h*_*w*_^*PS*^) + ∑_*k*_(1 – *y*_*A*, *H*_2_*O*_^*PS*^)*c*_*p*, *A*_(*T_MVR_* – *T*_*A*_^*PS*^)], where (*h*_*w*_^*MVR*^– *h*_*w*_^*PS*^) is the relative enthalpy of water between phase separation and the MVR unit, and *T_HTL_* is the operating temperature of the MVR unit. The electric power consumptions are calculated from *Ẇ*_*el*_^*MVR*^= *V̇*_*A*_^*HTL*^*w*_*el*_^*MVR*^, where *w*_*el*_^*MVR*^is the specific electric load per unit feed volume, assumed to be constant and equal to^27^ 39.8 kWh/m^3^.

### 3.4 Combustion of the HTL Gas

The analysis of the overall process in the boiler includes the mass and energy flows and composition of the flue gas at the boiler outlet, the mass and energy flows of combustion air, and the net thermal power transfer to the thermal fluid. The main input process parameters in the boiler are the mass flow rates of HTL gas and natural gas, denoted by *Ṁ*_*G*_^*HTL*^and *Ṁ*_*NG*_^*COMB*^, the excess air ratio for the combustion process λ_*g*_^*COMB*^defined as the ratio between the total inlet combustion air and the stoichiometric air, the inlet temperature for the combustion air *T*_*a*_^*COMB*^, and the flue gas temperature at the boiler outlet *T*_*g*_^*COMB*^. Natural gas consumption in the boiler is calculated from *Ṁ*_*NG*_^*COMB*^LHV_NG_= [(*Q*_*th*_^*HTL*^+ *Q̇*_*th*_^*MVR*^) – *Ṁ*_*G*_^*HTL*^*LHV*_*G*_^*HTL*^]/ϵ_*th*_, where ϵ_*th*_ is the thermal efficiency of the boiler defined as the output heat transferred to the thermal fluid relative to the input energy to the boiler from the HTL gas and the natural gas, which is assumed to be constant and equal to 0.9. The overall combustion process is calculated from steady-state mass and energy conservation equations and assuming the overall chemical reaction CH_*a*_N_*b*_O_*c*_S_*d*_ + ν_O_2__(O_2_ + 3.76N_2_) → CO_2_ + (*a*/2)H_2_O + ν_NO_NO + *d*SO_2_ + (3.76ν_O_2__ + *b*/2 – ν_NO_/2)N_2_. Here, CH_*a*_N_*b*_O_*c*_S_*d*_ represents the chemical formula for each of the combustible species *j* in the input fuel in the HTL gas and the natural gas, with *a*, *b*, *c*, and *d* representing the atomic molar composition of H, N, O, and S relative to C, and ν_O_2__ = 1 + *c*/2 – *a*/4 – *d* is the moles of air required for stoichiometric combustion of one mol of each combustible species. Then, the mass and energy flows of the combustion air at the boiler inlet can be written, respectively, as {*Ṁ*_*air*,_^*COMB*,^*Ḣ*_*air*_^*COMB*^} = ∑*_j_Ṁ*_*j*_^*COMB*^(*MW_air_*/*MW_j_*)ν_*O*_2_, *j*_(λ_*g*_^*COMB*^/*x*_*O*_2__^*air*^){1, *h*_*air*_^*COMB*^}, where *j* denotes each combustible species, ν_*O*_2_, *j*_ is the stoichiometric moles of oxygen required for complete combustion of one mol of *j*, *MW_air_* is the molecular weight of the air, and *h*_*air*_^*COMB*^is the molar enthalpy of air evaluated at *T*_*air*_^*COMB*^. The mass flow rate of the flue gas at the boiler outlet is calculated by applying a mass conservation equation to the entire boiler from *Ṁ*_*g*_^*COMB*^= ∑*_j_Ṁ*_*j*_^*COMB*^*m*_*g*, *j*_^*COMB*^, where *m*_*g*, *j*_^*COMB*^is the specific mass of flue gas produced from each combustible species, which is obtained from *m*_*g*, *j*_^*COMB*^= 1 + (*MW_air_*/*MW_j_*)ν_*O*_2_, *j*_(λ_*g*_^*COMB*^/*x*_*O*_2__^*air*^). The O_2_, N_2_, H_2_O, CO_2_, and SO_2_ compositions in the flue gas are calculated using the conservation equations for C, N, H, O, and S from
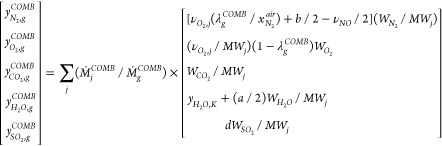
2

In this equation, it is assumed that all sulfur present in the flue gas is in the form of sulfur dioxide. The concentration of NO has been estimated to be 290 ppm based on experimental results^28^ from combustion of methane and CH_4_/NH_3_ mixtures in gas turbines, which shows a constant asymptotic value for NO concentration in the flue gas when the NH_3_ concentration in the gas fuel is above 5% vol. The energy flow rate of the flue gas at the boiler outlet is evaluated based on the mass flow rate and composition from *Ḣ*_*g*_^*COMB*^= ∑*_j_Ṁ*_*j*_^*COMB*^*m*_*g*, *j*_^*COMB*^*h*_*g*, *j*_^*COMB*^, where the specific enthalpy *h*_*g*, *j*_^*COMB*^is estimated from *h*_*g*, *j*_^*COMB*^= ∑*_j_y*_*j*, *g*_^*COMB*^(*h̅_j_*/*W_j_*), where *h̅_j_* is the molar thermal enthalpy for each species evaluated at *T*_*g*_^*COMB*^.

### 3.5 Flue Gas Cleaning after HTL Gas Combustion

Cleaning of the flue gas from the combustion process involves reduction of NOx and removal of SO_2_. The reduction of NOx is performed in a selective catalytic reduction (SCR) unit, the overall process performance being defined in terms of the NO removal efficiency η_SCR_, the total volumetric flow rate of ammonia consumed, *V̇*_*NH*_3__^*SCR*^= *V̇*_*g*_^*COMB*^*x*_*g*, *NOx*_^*COMB*^λ_*NH*_3__^*SCR*^, the consumption of catalyst *V̇*_*cat*_^*SCR*^, and the overall electric power consumption *Ẇ*_*el*_^*SCR*^= *V̇*_*g*_^*SCR*^*w*_*el*_^*SCR*^. Here, the parameter λ_*NH*_3__^*SCR*^represents the moles of ammonia consumed by the SCR per unit mol of NOx in the flue gas. The NOx reduction efficiency is assumed to be 92% at an operating temperature of 390 deg. C.^29^ The consumption of catalyst is evaluated considering a reference catalyst lifetime 32,000 h typically achieved in SCR units after natural gas boilers.^29^ The composition of the flue gas after the SCR is calculated assuming an overall NO reduction chemistry defined by the overall reaction NO + NH_3_ + (1/4)O_2_ → N_2_ + (3/2)H_2_O. Then, the flow rates of the different gas species leaving the SCR can be written as *V̇*_*G*, *i*_^*SCR*^= *V̇*_*G*, *i*_^*COMB*^for *i* ≠ H_2_O, NO, NH_3_, N_2_, and O_2_ and
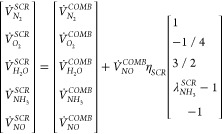
3

The total flue gas mass flow and its mass composition can then be written as *x*_*j*_^*SCR*^= *V̇*_*j*_^*SCR*^/*V̇*_*g*_^*SCR*^, with *V̇*_*g*_^*SCR*^= ∑*_j_V̇*_*j*_^*SCR*^. The reaction heat for the NO reduction is assumed to be small compared to the to the total thermal enthalpy of flue gas, and therefore, the gas temperature variation in the SCR have been neglected. The removal of SO_2_ in the dry scrubber (DS) is evaluated in terms of the consumption of lime *Ṁ*_*lime*_^*DS*^= λ_*lime*_^*DS*^*V̇*_*g*_^*SCR*^*x*_*SO*_2_, *g*_^*SCR*^, the electric power *Ẇ*_*el*_^*DS*^= *V̇*_*g*_^*SCR*^*w*_*el*_^*DS*^and the SO_2_ removal efficiency η_*SO*_2__^*DS*^The consumption of quicklime has been assumed to be proportional to the total inlet SO_2_ molar flow rates where λ_*lime*_^*WS*^is a fixed coefficient representing the unit mass of lime consumed per mol of SO_2_. The operating temperature in the dry scrubber is assumed to be 140 deg. C, above the dew point SO_2_. The overall chemistry in the dry scrubber is defined by the overall reaction SO_2_ + Ca(OH)_2_ → CaSO_3_ + H_2_O. It has been assumed that the CaSO_3_ and unreacted lime are separated from the flue gas stream in the bag filters. The mass flow of the different gas species leaving the scrubber are calculated from *Ṁ*_*g*, *i*_^*DS*^= *Ṁ*_*g*, *i*_^*SCR*^for *i* ≠ H_2_O, SO_2_, *Ṁ*_*g*, *H*_2_*O*_^*DS*^= *Ṁ*_*g*, *H*_2_*O*_^*SCR*^+ *Ṁ*_*lime*_^*DS*^*y*_*H*_2_*O*_^*lime*^and *Ṁ*_*g*, *SO*_2__^*DS*^= (1 – η_*SO*_2__^*DS*^)*Ṁ*_*SO*_2__^*SCR*^. The total flue gas mass flow and its mass composition can then be written as *Ṁ*_*g*_^*DS*^= ∑*_j_Ṁ*_*g*, *j*_^*DS*^and *y*_*g*, *j*_^*DS*^= *Ṁ*_*g*, *j*_^*DS*^/*Ṁ*_*g*_^*DS*^. Since the overall heat of the reactions between hydrated lime and acid gases is small in comparison to the heat absorbed by the evaporation of the excess water, then it has been neglected.

#### 3.5.1 Guard and Hydrotreating Reactor Processes

The overall processes in the guard and hydrotreating reactors are defined in terms of the mass yields *m*_*k*_^*S*^and composition *y*_*i*, *k*_^*S*^of the oil, gas, and aqueous phases at the reactor outlet and the required input hydrogen *Ṁ*_*H*_2__^*S*^. Here, the superscript *S* denote the guard (GR) or hydrotreating (HT) processes, the subscripts *k* denote the oil (O), gas (G), and aqueous (A) phases, and the subscript *i* denotes the atomic composition. The input hydrogen to each process *S* is calculated from *Ṁ*_*H*_2__^*S*^= *Ṁ*_*f*_^*S*^*m*_*H*_2_, *r*_^*S*^λ_*H*_2__^*S*^, where *m*_*H*_2_, *r*_^*S*^denote the hydrogen reacted per unit mass of input feed and λ_*H*_2__^*S*^is the ratio between the total hydrogen input and the reacted hydrogen. The overall conversion in the guard and hydrotreating processes has been evaluated semi-empirically throughout the so-called transfer coefficients *f*_*i*, *k*_^*S*^and *f*_*H*_2_, *k*_^*S*^defined, respectively, as the mass distribution of the atomic composition of the dry fraction of the input feed among the different phases produced and the distribution of the hydrogen reacted in the process among phases. From these definitions, the mass flow rate of the oil, gas, and aqueous phases are calculated from
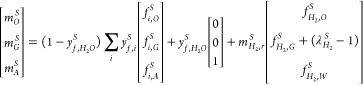
4

Similarly, the atomic composition can be evaluated from
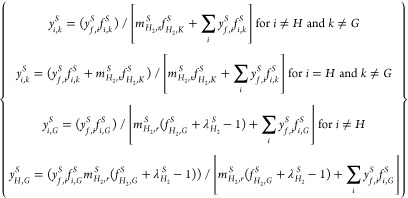
5

**Table 5:** Yields and Composition Measured for the Guard Reactor and Hydrotreating Reactor during Pilot Tests

test	biocrude	guard bed stage 1	guard bed stage 2	hydrotreating
operational conditions				
temperature (deg. C)		290	290	360
pressure (bar-a)		100	100	100
WHSV (1/h)		0.5	0.5	0.4
H2 reacted (% wt.)		0.36	0.17	1.57
yields (wt. %)				
liquid hydrocarbons		93.28	94.55	95.34
gas		4.20	2.80	3.81
water		2.52	2.65	0.85
oil composition (dry basis)				
carbon (wt. %)	76.84	79.54	79.94	83.68
hydrogen (wt. %)	9.15	10.41	10.73	11.90
nitrogen (wt. %)	3.82	3.80	3.74	2.86
sulfur (wt. %)	0.76	0.42	0.31	0.14
oxygen (wt. %)	9.43	5.83	5.28	1.42
iron (ppm)	1054	467	84	<10 ppm
gas composition (mol % H2 free)				
CH_4_		14.39	22.84	16.74
ethane		3.40	11.11	1.15
propane		1.13	5.75	0.77
butane		0.28	1.52	1.30
CO_2_		77.70	34.78	67.83
NH_3_		0.00	0.00	1.08
H_2_S		3.10	24.01	11.14

Table 6 shows the values of the transfer coefficients *f*_*i*, *k*_^*S*^and *f*_*H*_2_, *k*_^*S*^estimated using the measurements obtained during pilot-scale tests shown in Table 5.

**Table 6:** Estimated Values of the Transfer Coefficients for Dry Atomic Composition of the Feed *f*_*i*, *k*_^*S*^and the Reacted Hydrogen *f*_*H*_2_, *k*_^*S*^among Product Phases in the Overall Guard and Hydrotreating Processes

process	guard (overall)			hydrotreating		
phase	oil	gas	aqueous	oil	gas	aqueous
reacted H_2_	0.45	0.33	0.22	0.70	0.21	0.09
C (dry feed)	0.925	0.006	0.069	0.899	0.006	0.096
H (dry feed)	0.987	0.012	0.002	0.924	0.007	0.069
O (dry feed)	0.498	0.028	0.474	0.656	0.005	0.338
N (dry feed)	0.871	0.000	0.129	0.388	0.464	0.148
S (dry feed)	0.363	0.314	0.323	0.231	0.128	0.641
Fe (dry feed)	0.56			0.82		

#### 3.5.2 Hydrocracking

The overall hydrocracking processes have been defined in terms of the total input and reacted mass of hydrogen, denoted, respectively, by *m*_*H*_2__^*HC*^and *m*_*H*_2_, *r*_^*HC*^per unit feed mass, the mass yields of main phase products *m*_*k*_^*HC*^with *k* denoting liquid oil, gas, and process water. Table 8 shows the values of *m*_*H*_2__^*HC*^, *m*_*H*_2_, *r*_^*HC*^, and *m*_*k*_^*HC*^used in the analysis, which are based on reported data on hydrocracking of heavy distillate derived from distillation of hydrotreated pyrolysis oil.^30^ To the knowledge of the authors, there are no experimental or simulation results in the literature on hydrocracking of heavy distillates produced from hydrotreating of sewage sludge-derived HTL oils. Therefore, although the composition of heavy distillates derived from pyrolysis oil and HTL oil can differ significantly, the approach of using pyrolysis oil results has been considered here just to obtain estimates of the product yields and the consumption of hydrogen and catalysts. It has been assumed that all the remaining S and N heteroatoms present in the heavy distillation feed to hydrocracking are reduced to H_2_S and NH_3_, with mass yields calculated from *m*_*H*_2_*S*_^*HC*^= (*y*_*S*, *f*_^*HC*^/*MW_S_*)*MW*_*H*_2_*S*_ and *m*_*NH*_3__^*HC*^= (*y*_*N*, *f*_^*HC*^/*MW_N_*)*MW*_*NH*_3__, where *y*_*i*, *f*_^*HC*^denotes the atomic composition of the heavy distillate feed to the hydrocracking process.

#### 3.5.3 Distillation

**Figure 3:**
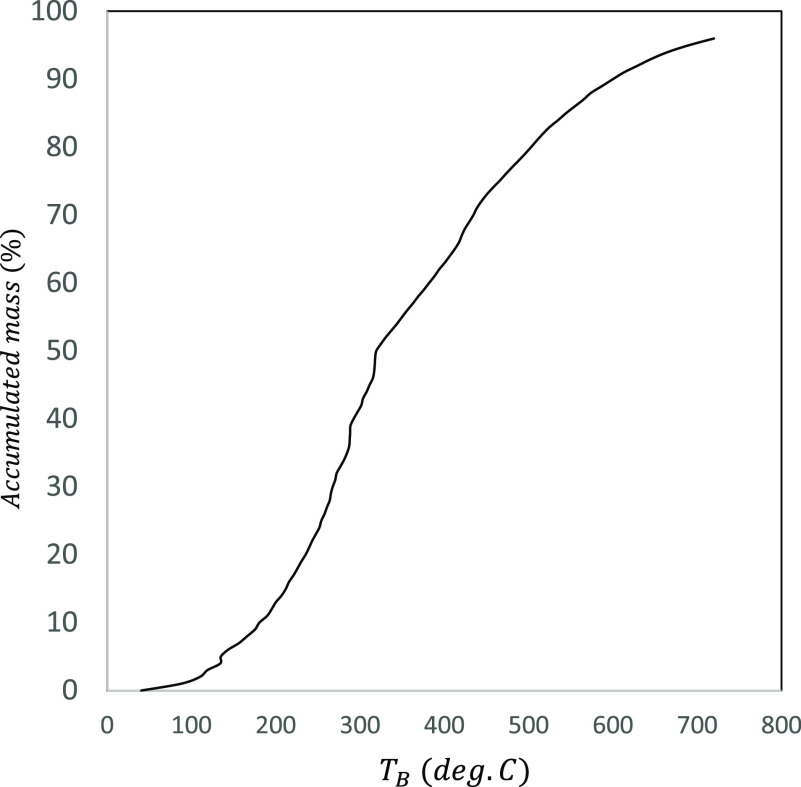
Measured distillation curve for the oil produced hydrotreating of HTL biocrude during pilot tests by Steeper Energy.

The distillation fractions considered in the analysis with specification of the boiling temperature range reference values for the carbon numbers, specific gravity, carbon and hydrogen content, average molecular weight, and high heating value are shown in Table 7. The overall distillation of the mixture of the liquid oil products from the hydrotreating and hydrocracking process is defined in terms of the mass flow rates of the input feed and the distillation products, calculated, respectively, from *Ṁ*_*j*_^*DIST*^= *Ṁ*_*O*_^*HT*^+ *Ṁ*_*O*_^*HC*^and *Ṁ*_*j*_^*DIST*^= *Ṁ*_*O*_^*HT*^*y*_*j*_^*HT*^+ *Ṁ*_*O*_^*HC*^*y*_*j*_^*HC*^. Here, the subscript *j* represents each of the distillation fractions, i.e., light hydrocarbon gases, naphtha, middle distillate, and heavy distillate, and *y*_*j*_^*HT*^and *y*_*j*_^*HC*^are the mass fractions of *j* in the hydrotreating and hydrocracking oils shown in Table 8. The values of *y*_*j*_^*HT*^are shown in Table 7 calculated from *y*_*j*_^*HT*^= ∫_*TB*_*j*, *min*__^*TB*_*j*, *max*_^*f*_*O*_^*HT*^(*T_B_*)*dT_B_*, which represents the integral of the measured distillation curve *f*_*O*_^*HT*^(*T_B_*), shown in Figure 3, over the range of boiling temperatures specified by each distillation fraction, as shown in Table 8. The values of *y*_*j*_^*HC*^in Table 7 are based on literature data.^30^

**Table 7:** Definition and Reference Properties of the Distillation Fractions Considered in the Analysis

distillation fraction	light hydrocarbons	naphtha	middle distillate	heavy distillate
TBP (deg. C)	<80	80–210	210–340	<340
carbon number	<C_5_	C_6_–C_10_	C_11_–C_20_	>C_20_
specific gravity	0.66	0.78	0.82	0.94
carbon (% wt.)	82.43	84. 5	86.21	87.1
hydrogen (% wt.)	16.1	14.2	13.5	12.9
molecular weight (g/mol)	102	130	200	425
HHV (MJ/kg)	48.5	46.7	45.8	44.3

**Table 8:** Total Mass of Hydrogen Input and Reacted and Mass Yields of Products per Unit Feed Mass for (1) Processing HTL Biocrude in a Guard Reactor and Hydrotreater Based on Experimental Results and (2) Hydrocracking of Heavy Distillate Derived from Distillation of Hydrotreated Pyrolysis Oil^30^

process	guard reactor and hydrotreating	hydrocracking
reacted hydrogen (% wt.)	19.2	5.4
light hydrocarbons yield (% wt.)	1.25	11.5
naphtha yield (% wt.)	13.55	26.9
middle distillate yield (% wt.)	29.9	61.6
heavy distillate yield (% wt.)	55.3	

#### 3.5.4 Amine Gas Treatment

The amine system is considered as a package defined in terms of the total amine flow rate required in the absorber *V̇*_*MEA*_^*ABS*^and the specific heating *Q̇*_*h*_^*GT*^, cooling *Q̇*_*c*_^*GT*^, and electricity *Ẇ*_*el*_^*GT*^required per unit mass of amine injected into the absorber. The mass flow rate of amine to the absorber is calculated from *V̇*_*MEA*_^*ABS*^= γ[ *V̇*_*CO*_2__^*CC*^/χ_*CO*_2__^*ABS*^+ *V̇*_*H*_2_*S*_^*CC*^/χ_*H*_2_*S*_^*ABS*^+ *V̇*_*NH*_3__^*CC*^/χ_*NH*_3__^*ABS*^]/*P*_*g*_^*ABS*^, where χ_*j*_^*ABS*^are the solubility of CO_2_, H_2_S, and NH_3_ in MEA at the absorber temperature and γ is the ratio of the actual flow ratio of amine relative the flow rate required to reach equilibrium. Here, we have considered a linear dependence for the solubility. The total heating, cooling, and electric power demands by the overall amine system have been assumed to be proportional to the flow rate of amine to the absorber and are calculated from [*Q̇*_*h*_^*GT*^, *Q̇*_*c*_^*GT*^, *Ẇ*_*el*_^*GT*^] = *V̇*_*MEA*_^*ABS*^[*q*_*h*_^*GT*^, *q*_*c*_^*GT*^, *w*_*el*_^*GT*^], where *q*_*h*_^*GT*^, *q*_*c*_^*GT*^, *w*_*el*_^*GT*^are the specific heating, cooling, and electricity required per unit volume of amine, assumed to be constant and equal to,^31^ respectively, 0.27 MJ/kg, 0.40 MJ/kg, and 46.5 kWh/kg.

### 3.6 Material and Energy Balances for the Overall Conversion of Biofuels from Sewage Sludge

The main material and energy flows for the production and upgrading of HTL biocrude from sewage sludge are shown, respectively, in Tables 9 and 10. The conversion of sewage sludge to biocrude exhibits a conversion efficiency of 73.4% on energy basis and 29.4% on dry-mass basis. The overall mass and energy yields of combined naphtha and middle distillate from sewage sludge on dry basis are approximately 19 and 60%, where the naphtha fraction represents about 45% of the total. Losses in the chemical energy during the hydrothermal liquefaction of the sewage sludge are in the form of dissolved organic components in the aqueous phase, short-chain hydrocarbons in the gas phase, and unconverted non-dissolved carbon in the solid residue, which represent about 17, 4.9, and 4.6% on energy basis, respectively. Chemical energy from the biocrude, which are not converted to naphtha and middle distillate, are mainly in the form of light hydrocarbon gases and dissolved organics on the process water after hydrotreating and hydrocracking, which represent about 11.7% and 10% of the biocrude energy. The net heat demand in the production of biocrude represents approximately 20% of the total feedstock energy, of which 8% is covered by combustion of the HTL gas and stripped ammonia and the remaining 12% by an external source of natural gas. The overall heat demand by the complete upgrading process is 4.9% of the chemical energy content in the biocrude, of which 1.2% is used by hydrotreating, 1.3% by hydrocracking, and 2.4% by distillation. The total hydrogen consumed in the overall upgrading is approximately 4.0% wt. relative to the biocrude feed. The distribution of hydrogen consumption between the hydrotreating, including the guard reactor, and the hydrocracking processes is approximately the same. The CO_2_ emissions by the overall sludge to the biofuel conversion process are about 0.58 kg CO_2_ per dry kg sewage sludge, of which 64% is biogenic from combustion of HTL gases and light hydrocarbons produced during upgrading and 36% is fossil-based from combustion of natural gas for covering the heat demand of the biocrude production and from reforming of natural gas for production of the make-up hydrogen used in the upgrading.

**Table 9:** Main Material Flows Based on an Input Feed of 1 Dry Ton Sewage Sludge

sewage sludge	ton	4.29
slurry to HTL	ton	5.44
HTL oil	ton	0.29
HTL aqueous phase	ton	3.96
HTL solid	ton	1.03
HTL gas	ton	0.16
base (NaOH) to hydrothermal liquefaction	kg	14.5
catalyst (K_2_CO_3_) to hydrothermal liquefaction	kg	6.2
citric acid to phase separation	kg	5.0
MEK to phase separation	kg	
combustion air	Nm^3^	879
natural gas	kg	35.2
lime (gas cleaning)	kg	4.04
flue gas	ton	1.33
flue gas	Nm^3^	1593.8
CO_2_ to air	ton	0.35
fossil	ton	0.16
biogenic	ton	0.19
NH_3_ to SCR	kg	0.69
catalyst to SCR	kg	0.14
dry scrubber residue	kg	4.04
MVR concentrate bleed to disposal	ton	0.28
process water from biocrude production	ton	3.96
emissions to water from biocrude production plant	dm^3^	2.07
light HC from stripping	kg	14.55
organic liquid to distillation	ton	0.316
light HC from distillation	kg	14.6
naphtha range from distillation	kg	85.8
diesel range from distillation	kg	100.3
heavy fraction from distillation	kg	115.2
guard reactor catalyst	kg	0.046
hydrotreating catalyst	kg	0.037
hydrocracking catalyst	kg	0.018
process water	dm^3^	0.031
sour gas from separation	kg	30.19
total make-up H_2_	kg	11.87
consumption in guard reactor	kg	1.52
consumption in hydrotreating	kg	4.13
consumption in hydrocracking	kg	6.22
total fuel gas consumption	kg	8.59
to fire heater before hydrotreating	kg	3.25
to fire heater before distillation column	kg	1.72
to fire heater before hydrocracking	kg	3.62
make-up amine consumption	kg	0.11
fresh water	dm^3^	2.06
GHG emissions	kg CO_2.eq_	576.5
from combustion of natural gas	kg CO_2.eq_	157.6
from combustion of HTL gases	kg CO_2.eq_	188.8
from make-up H_2_ production	kg CO_2.eq_	83.7
from light HC combustion (fired heaters)	kg CO_2.eq_	146.4
solid residue	kg	0.10

**Table 10:** Main Energy Flows (MW) Based on an Input Feedstock Chemical Energy of 1 MW Based on HHV

chemical energy raw sludge	1
slurry to HTL	1.17
chemical energy oil product	0.734
chemical energy aq. effluent after phase separation	0.385
chemical energy gas after phase separation	0.049
chemical energy solid residue after phase separation	0.046
chemical energy MVR concentrate bleed to disposal	0.066
chemical energy treated water to disposal	0.01
heating slurry preparation	0.103
heating slurry to HTL	0.457
heat recovery from HTL product cooling	0.418
heating of HTL process water before MVR	0.009
MVR condensate cooling	0.044
natural gas consumption	0.12
heat recovery boiler	0.126
heat recovery after SCR	0.034
heat lost from flue gas to air	0.011
chemical energy biocrude feed	0.734
chemical energy light gases	0.074
chemical energy naphtha	0.281
chemical energy middle distillate	0.321
chemical energy H_2_ consumed	0.027
heating biocrude before hydrotreating	0.009
heating organic liquid before distillation	0.017
heating organic liquid before hydrocracking	0.010

## 4 Equipment Scale-Up and Cost Analysis

The economic performance of the overall production and upgrading of the biocrude has been evaluated as a function of the dry sludge feed capacity to the biocrude production and the biocrude feed capacity to the upgrading, denoted respectevely by *Ṁ*_*S*_^*HTL*^and *Ṁ*_*BC*_^*UPG*^. This analysis involves scale-up of the equipment included in the process flow diagrams shown in Figures 1 and 2 using parametric models for the equipment design and costs described bellow. It has been assumed that the feedstock composition and all the process design parameters shown in Tables 1 and 2 are constant for the whole range of plant capacity and that the variations of all mass and enthalpy flow rates have a linear dependency on the conversion capacity. The capacity range used for the biocrude upgrading is 7 to 70 ton/day, which corresponds to the biocrude produced from sewage sludge with a feed capacity of 30–300 dry ton/day based on a biocrude conversion efficiency of approximately 29.4%.

### 4.1 Equipment Design Models

The size of silos and liquid storage tanks are specified by their volume *V*, calculated from *V* = *Ṁ_f_t_R_*φ/(1 – ϕ), as a function of the feed mass flow rate *Ṁ*_*f*_ and density ρ_*f*_ and the residence time *t_R_*. The parameters ϕ and φ denote, respectively, the porosity of the bed material and the empty volume to material filled volume ratio. For all storage equipment, the value of φ is constant and equal to 1.2. Conveyors are defined in terms of the length *L* and diameter *D*. The diameter is calculated from *D* = [(*Ṁ*_*f*_/ρ_*f*_)/*v_f_*]^1/2^ based on the characteristic velocity of the feed along the conveyor *v_f_*, assumed to be constant and equal to 5 cm/s. The length of the conveyor has been estimated considering the layout distance between equipment. The total electric driving power is calculated from *Ẇ*_*el*_ = (*Ẇ*_*N*_ + *Ẇ*_*M*_ + *Ẇ*_*H*_)/η_*m*_, where *Ẇ*_N_ = *DL*/20 is the empty power loss, *Ẇ*_*M*_ = *f*_m_*gṀ_f_L* is the load power due to friction losses caused by the weight of the material, and *Ẇ*_*H*_ = *gṀ_f_H* is the power required to overcome an elevation difference *H*. Here, the constant *f_m_* is a progress resistance representing an artificial friction coefficient for the moving material, *g* is the gravitational constant, and η_*m*_ is the electric to mechanical power efficiency of the motor assumed to be constant and equal to 0.9. Pumps and fans are specified by the total electric power, calculated from *Ẇ*_*el*_ = *Ṁ_f_gH_p_*/η_*m*_, where η_*m*_ is the electric to mechanical power efficiency of the motor and *H_p_* = (Δ*P* + *ΔP_p_* + *ΔP_eq_*)/ρ*g* + d*z* represents the total head. Here, *ΔP* is the pressure increase required by the main downstream process, *ΔP_p_* and *ΔP_eq_* are the pressure losses in the piping and auxiliary equipment between the pump or fan and the downstream process equipment, respectively, and d*z* is the difference in elevation. This formulation assumes that the variations of the kinetic energy due to reduction or increase in flow velocities are negligible. Also, the effect of the elevation in the pressure drop when calculating fans has been neglected. Piping losses have been calculated from *ΔP*_p_ = (*K*_p_ + *f*_p_*L*_p_/*D*_p_)ρ*v*^2^/2, where *v*, ρ, *L_p_*, and *D_p_* denotes, respectively, the internal fluid velocity, the fluid density, the pipe length, and the pipe diameter. The parameters *f_p_* and *K_p_* represent the friction coefficient for the fully developed internal flow and the pressure drop coefficients due to elbows, valves, and fittings. The friction coefficient *f_p_* is calculated as a function of the Reynolds number *Re* = ρ*vD_p_*/μ and the pipe roughness number ε, using Poiseuille’s law *f_p_* = 64/*Re* for laminar flows (*Re* < 2300) and the Colebrook–White correlation^32^, with  for turbulent flows (*Re* < 4000). The characteristic piping length is a function of the equipment layout and thus the plant capacity. Compressors have been specified based on the total electric power calculated from *Ẇ*_*el*_ = (*Ṁ*_*g*_/ρ_*g*_)*P_i_*[(*P_o_*/*P_i_*)^(1 – 1/*k*)^ – 1][*k*/(*k* – 1)]/η_*m*_, where *Ṁ*_*g*_ and ρ_*g*_ are the mass flow rate and density of the gas, *P_i_* and *P_o_* are the inlet and discharge pressure, η_m_ is the electric to mechanical power efficiency of the motor, and *k* is the polytropic coefficient assumed to be constant and equal to 0.8. Specification of the heat exchangers has been evaluated based in the total duty *Q̇*_*th*_ and heat transfer area *A_th_*. Calculations of the duty depends on whether the heat exchanger is used as a cooler or heater. For a heater, the duty *Q̇*_*th*_ = *Ṁ_h_c*_*p*, *h*_(*T*_*h*_^*in*^– *T*_*h*_^*out*^) is a function of the inlet and outlet temperatures, the mass flow rate, and the specific heat capacity of the hot stream. Likewise, the duty for a cooler is calculated from *Q̇*_*th*_ = *Ṁ_c_c*_*p*, *c*_(*T*_*c*_^*in*^– *T*_*c*_^*out*^), where the subscript denotes here the cold stream. Based on the duty, the total heat transfer area is calculated from *A_th_* = *Q̇*_*th*_/(*U_th_LMTD*), where *LMTD* is the log mean temperature difference and *U_th_* is the overall heat transfer coefficient. It has been assumed that all heat exchangers are designed as a shell and tube in counterflow, with the overall heat transfer coefficient calculated from *U_th_* = {*d_t_*/*Nu_t_k_t_* + (*d_t_*/2*k_t_*)*Ln*[*d_t_*/(*d_t_* – 2*t*)] + (*d_t_* – 2*t*)/*Nu_s_k_s_*}^−1^. In this equation, *Nu_t_*, *k_t_*, *Nu_s_*, and *k_s_* are the Nusselt number and the thermal conductivity for the flows in the tubes and shell, respectively, and *d_t_*, *t*, and *k_t_* are the tube diameter, thickness, and thermal conductivity. Assuming horizontal staggered tubes, the Nusselt numbers for the shell can be estimated from . The Nusselt number for fluids inside the tubes are calculated using correlation for internal flows in cylindrical tubes *Nu*_*d*_*t*__^*B*^= 0.023*Re*_*t*_^4/5^*Pr*^0.3^. Stirred tanks are specified by the total volume and dimensions of the tank and the total electric power of the impeller. The tank volume is calculated from *V_T_* = (*Ṁ*_*f*_/ρ_*f*_)*t_R_* in terms of the residence time *t*_R_ and the feed mass flow rate *Ṁ*_*f*_ and density ρ_*f*_. All stirred tanks are assumed to be cylindrical with the diameter and height calculated from *D* = (4*V_T_*/π*k_HD_*)^1/2^ and *L* = *k_HD_*(4*V_T_*/π*k_HD_*)^1/2^, where *k_HD_* is the height to diameter ratio, which is assumed to be constant and equal to 2. The power consumption is calculated from *Ẇ*_*el*_^*SR*^= *N_P_*ρ*N*_*I*_^3^*D*_*I*_^5^/η_*el*_^*M*^, where *N_l_* is the impeller rotational speed (rpm), *D*_I_ is the impeller diameter, and *N*_P_ = 346.7/*Re_I_* + 1.27 is the impeller power number based on the Reynolds number *Re_I_* = (*N_I_*/60)*D*_*I*_^2^ρ_*f*_/μ_*f*_. The filter press is modeled after the plate and frame press type with a constant filtration rate and an applied pressure differential *ΔP* = (*V̇*/*A_c_*)*t_c_*ηα_*c*_, where α_*c*_ = 1/κ_*c*_ is the filter cake resistivity, κ_*c*_ is the permeability, and η is the viscosity. The filter cake resistivity α_*c*_ = *f*(*d_h_*) is a function of the hydraulic pore diameter *d_h_* = 4ϕ_*c*_/[(1 – ϕ_*c*_)*S_V_*] of the cake through the Kozeny–Carman Equation α_*c*_ ∼ 5(1 – ϕ_*c*_)^2^/(ϕ*_c_S_V_*)^2^. Here, *S_V_* is the inner solid surface area per unit volume, which for spherical particles of an average diameter (*d_s_*) is equal to *S_V_* = 6/*d_s_*. A centrifuge is described in terms of the total electric power, calculated from *Ẇ*_*el*_ = *k_w_*(*Ṁ*_*f*_/ρ_*f*_), where *k_w_* is the specific electricity consumption per unit volume of the input feed, assumed to be constant and equal to 1.4 kWh/m^3^. The size of all catalytic reactors is specified by their volume *V*, calculated as a function of the feed mass flow rate *Ṁ*_*f*_ and the weight hourly space velocity *WHSV*_*cat*_^*K*^specified for the catalyst from *V* = (*Ṁ*_*f*_^*K*^/*WHSV*_*cat*_^*K*^)ρ_*cat*_^–1^φ/(1 – ϕ), where ρ_*cat*_ is the density of the catalyst and the parameters ϕ and φ denote, respectively, the porosity of the bed material and the empty volume to material filled volume ratio. For all catalytic reactors, the values of ϕ and φ are constant and equal to 0.15 and 1.4, respectively.

### 4.2 Electric Power Loads

The calculated values of the electric power load for the biocrude production and upgrading as a function of the feed capacity are shown in Tables 11 and 12. The electric power consumption for all the main systems involved in the overall conversion process behaves almost linearly with the feed capacity, indicating that the effect of the higher pressure lost with larger plant layouts is small in the overall power consumption. The highest contributions to the power consumption in the biocrude production plant correspond to the MVR unit, the slurry pump, and the centrifuges in the phase separation systems, accounting approximately to 55, 26, and 15% of the total. The total electric load for the overall upgrading process is small, about 2%, compared to the biocrude production, the main contributions corresponding to the feeding pumps to the hydrotreating and hydrocracking processes, the amine system, and the hydrogen compressor, which account for 35.9, 14.7, 12.5, and 10% of the total.

**Table 11:** Electric Loads (kW) as a Function of the Plant Capacity for the Overall Production of Biocrude from Sewage Sludge

sewage sludge capacity (dry-ton/day)	30	50	100	150	200	250	300
slurry prep. and HTL	85.74	144.17	293.78	447.64	605.20	766.11	766.11
sludge pump	0.550	0.807	1.365	1.862	2.324	2.761	2.761
feedstock conveyor	1.354	2.349	5.090	8.110	11.351	14.778	14.778
slurry preparation stirred tank	1.947	4.558	14.456	28.402	45.866	66.519	66.519
catalyst conveyor	0.041	0.064	0.115	0.163	0.209	0.254	0.254
base conveyor	0.068	0.106	0.192	0.273	0.351	0.428	0.428
slurry pump	81.776	136.288	272.561	408.832	545.101	681.368	681.368
phase separation	57.29	95.48	190.97	286.45	381.93	477.41	477.41
first centrifuge	32.446	54.076	108.152	162.228	216.304	270.380	270.380
mixing vessel after centrifuge	4.563	7.605	15.211	22.816	30.421	38.027	38.027
second centrifuge	20.281	33.802	67.603	101.405	135.206	169.008	169.008
gas treatment	14.19	23.03	44.97	66.67	88.21	109.64	109.64
combustion air compressor	1.078	1.749	3.420	5.072	6.713	8.346	8.346
natural gas supply	0.174	0.276	0.523	0.762	0.997	1.230	1.230
exhaust fan	5.013	7.892	15.038	21.995	28.844	35.619	35.619
SCR package	2.814	4.690	9.381	14.071	18.761	23.451	23.451
dry scrubber package	4.573	7.622	15.243	22.865	30.487	38.109	38.109
lime conveyor	0.097	0.149	0.266	0.374	0.476	0.575	0.575
filter dust conveyor	0.100	0.154	0.275	0.387	0.493	0.595	0.595
thermal fluid pump	0.339	0.503	0.828	1.141	1.435	1.715	1.715
water treatment	200.04	333.32	666.45	999.54	1332.61	1665.66	1665.66
process water pump	1.058	1.738	3.417	5.082	6.737	8.387	8.387
MVR package	198.658	331.096	662.193	993.289	1324.385	1655.482	1655.482
CW pump condensate cooler	0.092	0.134	0.223	0.302	0.374	0.442	0.442
condensate pump	0.208	0.317	0.550	0.777	0.994	1.206	1.206
concentrate pump	0.025	0.037	0.065	0.091	0.116	0.140	0.140

**Table 12:** Electric Loads (kW) as a Function of the Plant Capacity for the Overall Production of Naphtha and Middle Distillate from Biocrude

sewage sludge capacity (dry-ton/day)	30	50	100	150	200	250	300
hydrotreating	1.978	3.236	6.342	9.413	12.479	15.523	18.558
biocrude pump	1.402	2.332	4.655	6.977	9.311	11.636	13.961
natural gas compressor	0.043	0.064	0.115	0.160	0.202	0.243	0.282
combustion air fan	0.210	0.333	0.629	0.915	1.195	1.472	1.746
exhaust fan	0.323	0.507	0.943	1.362	1.770	2.172	2.569
separation and fractionation	0.059	0.098	0.197	0.295	0.394	0.492	0.590
naphtha column	0.030	0.049	0.098	0.148	0.197	0.246	0.295
diesel column	0.030	0.049	0.098	0.148	0.197	0.246	0.295
hydrocracking	0.985	1.624	3.209	4.785	6.357	7.926	9.492
heavy distillate pump	0.574	0.951	1.894	2.836	3.777	4.718	5.659
fuel gas compressor	0.020	0.032	0.059	0.085	0.111	0.136	0.161
combustion air fan	0.160	0.263	0.518	0.771	1.023	1.274	1.524
exhaust fan	0.232	0.378	0.738	1.094	1.447	1.798	2.147
gas treatment and hydrogen recycle	0.854	1.351	2.511	3.646	4.729	5.820	6.898
amine system	0.487	0.811	1.623	2.434	3.246	4.057	4.869
H_2_-rich recycle compressor	0.221	0.325	0.528	0.722	0.903	1.074	1.239
make-up H_2_ compressor	0.146	0.214	0.361	0.490	0.580	0.688	0.791

### 4.3 Capital Cost

The capital cost has been evaluated in terms of the total permanent investment *C_TPI_*, calculated from
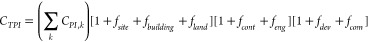
6

The first term in this formula represents the sum of the purchase and installation cost of each equipment included in the process design shown in Figures 1 and 2, calculated from *C*_*PI*, *k*_ = *C*_*P*, *k*_^*B*^(*S_k_*/*S*_*k*_^*B*^)^*n_k_*^(*I*/*I_B_*)*f*_*inst*, *k*_, where *C*_*P*, *k*_^*B*^and *S*_*k*_^*B*^are the base-case equipment purchase cost and equipment size, *S_k_* is the actual size of equipment, *n_k_* is the equipment scale factor, *f*_*inst*, *k*_ is the equipment installation factor, and *I*/*I_B_* is the price index ratio between the actual year and the reference year where the base case purchase cost function evaluated based on the Chemical Engineering Plant Cost Index (CEPCI). Table 13 lists the values for the equipment cost parameters used in the analysis. All the plant costs are updated to 2021. The parameters *f_i_* in eq 6 are additional capital cost factors associated with land, civil work for site preparation and construction of buildings, engineering and contingencies for civil work and process equipment, and project development and licenses. Representative values^33,36^ for *f_i_* are listed in Table 14.

**Table 13:** Parameters for Calculating the Purchase and Installation Cost for the Equipment

equipment	base specification		base purchase cost	installation factor	scale factor	base year	ref
screw conveyor	33.5	t/h	0.350	2.10	0.80	2002	(34)
belt conveyor	28.6	t/h	0.070	2.10	0.80	2009	(34)
sludge pump	45.0	kWe	0.175	2.47	0.70	2017	(35)
sludge tank	76.7	m^3^	0.174	2.47	0.70	2010	(35)
shell & tube heat exchanger	7.8	MW	0.080	1.24	0.60	2010	(34)
solid storage silo	2821.0	kg/h	0.055	2.10	0.70	2013	(34)
HTL slurry pump	333.0	kW	0.470	2.84	0.80	2011	(37)
HTL slurry preheater	17.5	MW	1.970	2.72	0.70	2012	(37)
HTL reactor	5.4	m^3^	0.270	2.47	1.00	2013	(37)
product cooler	74.9	MW	5.540	2.72	0.70	2013	(37)
gas separator	12.0	l/s	0.190	2.73	0.84	2007	(34)
flash tank	20.0	m^3^	0.014	2.73	0.71	2007	(34)
condenser	0.2	MW	0.067	1.67	0.53	2017	(34)
centrifuge	500	kg/h	0.149	2.47	0.38	2007	(34)
gas burner	1.0	MW	0.250	1.79	0.74	2007	(34)
syngas injector	0.0	kg/s	0.008	1.24	0.32	2007	(34)
natural gas burner	0.5	MW	0.002	3.03	0.16	2007	(34)
gas compressor	15.0	kW	0.014	2.47	0.70	2017	(34)
natural gas storage tank	20.0	m^3^	0.014	2.97	0.71	2007	(34)
exhaust fan	109.2	kW	0.600	2.47	0.70	2008	(34)
SCR (package)	53362.5	Nm^3^/h	0.833	2.47	0.70	2001	(37)
water pump	3.7	kWe	0.009	2.84	0.80	2013	(34)
scrubber + bag filter	53362.5	Nm^3^/h	2.054	2.47	0.70	2001	(37)
cylindrical atmospheric tank	1.5	m^3^	0.017	2.73	0.93	2007	(34)
cooler	0.4	MW	0.060	1.67	0.53	2017	(34)
MVR package	0.4	m^3^/h	0.490	2.47	0.50	2018	(27)
acid storage tank	1981	kg/h	0.100	1.73	0.71	2020	(34)
acid pump	1981.0	kg/h	0.023	2.47	0.70	2010	(34)
mixing vessel with agitator	500	kg/h	0.016	2.22	0.70	2019	(34)
oil tank	76.7	m^3^	0.174	2.47	0.70	2010	(30)
oil feed pump	8.5	l/s	0.104	4.42	0,8	2013	(30)
guard reactor	20	m3	0.231	2.73	0,81	2010	(30)
hydrotreating reactor	24,948	kg/h	6.05	1.77	1	2013	(30)
HP separator (3-phase)	9.15	kg/s	0.44	1.24	0.84	2007	(30)
LP stripper	0.924	kg/s	0.065	2.10	0.8	2017	(30)
naphtha column	6.62	kg/s	0.49	2.08	0.7	2013	(30)
naphtha condenser	4.08	kg/s	0.031	5.66	0.7	2013	(30)
naphtha accumulator	4.08	kg/s	0.125	4.95	0.7	2013	(30)
naphtha reflux pump	4.08	kg/s	0.032	5.32	0.8	2013	(30)
naphtha column reboiler	2.94	kg/s	0.048	3.49	0.7	2013	(30)
diesel column	2.94	kg/s	0.30	2.36	0.7	2013	(30)
diesel condenser	1.49	kg/s	0.013	8.23	0.7	2013	(30)
diesel accumulator	1.49	kg/s	0.031	5.36	0.7	2013	(30)
diesel reflux pump	1.49	kg/s	0.007	4.90	0.8	2013	(30)
diesel column reboiler	1.11	kg/s	0.027	3.54	0.7	2013	(30)
heavy distillate pump	1.1	kWe	0.046	2.47	0.7	2017	(30)
hydrocracking reactor	10,886	kg/h	2.62	2.43	1	2013	(30)
sour gas cooler	0.4	MW	0.060	1.67	0.53	2017	(30)
amine (package)	41.9	kg/s	5.45	2.69	0.65	2005	(31)
hydrogen compressor	15.0	kW	0.014	2.47	0.70	2017	(30)
fired heater	0.2	MW	0.143	1.88	0.6	2013	(30)

**Table 14:** Average Unit Prices for Consumables and Utilities and Financial Assumptions Considered for Calculating the Operating Costs and the Minimum Fuel Selling Price

direct operational cost	unit cost
gate fee sludge treatment, €/ton	250
sulfuric acid, €/ton	62
citric acid, €/ton	640
NaOH, €/ton	300
hydrothermal liquefaction catalyst (K_2_CO_3_), €/ton	1400
enzyme (protease), €/ton	1240
MEK, €/ton	1440
lime, €/ton	120
MgO, €/ton	150
NH_3_, €/liter	44.8
catalyst guard reactor, €/liter	31.4
catalyst hydrotreating, €/liter	31.4
catalyst hydrocracking, €/liter	31.4
solid residue disposal, €/ton	40
process water disposal, €/m^3^	8.3
fresh water, €/m^3^	0.5
electricity, €/kWh	1.0
natural gas, €/MWh	24
H_2_ production cost (SMR), €/kg	3.75
CO_2_ emissions (fossil), €/ton	25
amine (MEA), €/kg	2.9
labor average annual income, k€/year	
managers	162
O&M manager	88
engineers	96
maintenance technician	59
shift supervisor	66
shift operators	59
administration	37
site and building maintenance	37
overhead factor (operators only), %	20
labor overhead charge rate fraction	1.25
administration cost, % total permanent investment	2
insurance cost, % of the total permanent investment	1
loan interest rate, %	7
return of investment, %	10
equity to debt ratio	30/70
plant lifetime, years	25
construction time, years	2
commissioning time, years	1
annual operating time, h	8000

**Figure 4:**
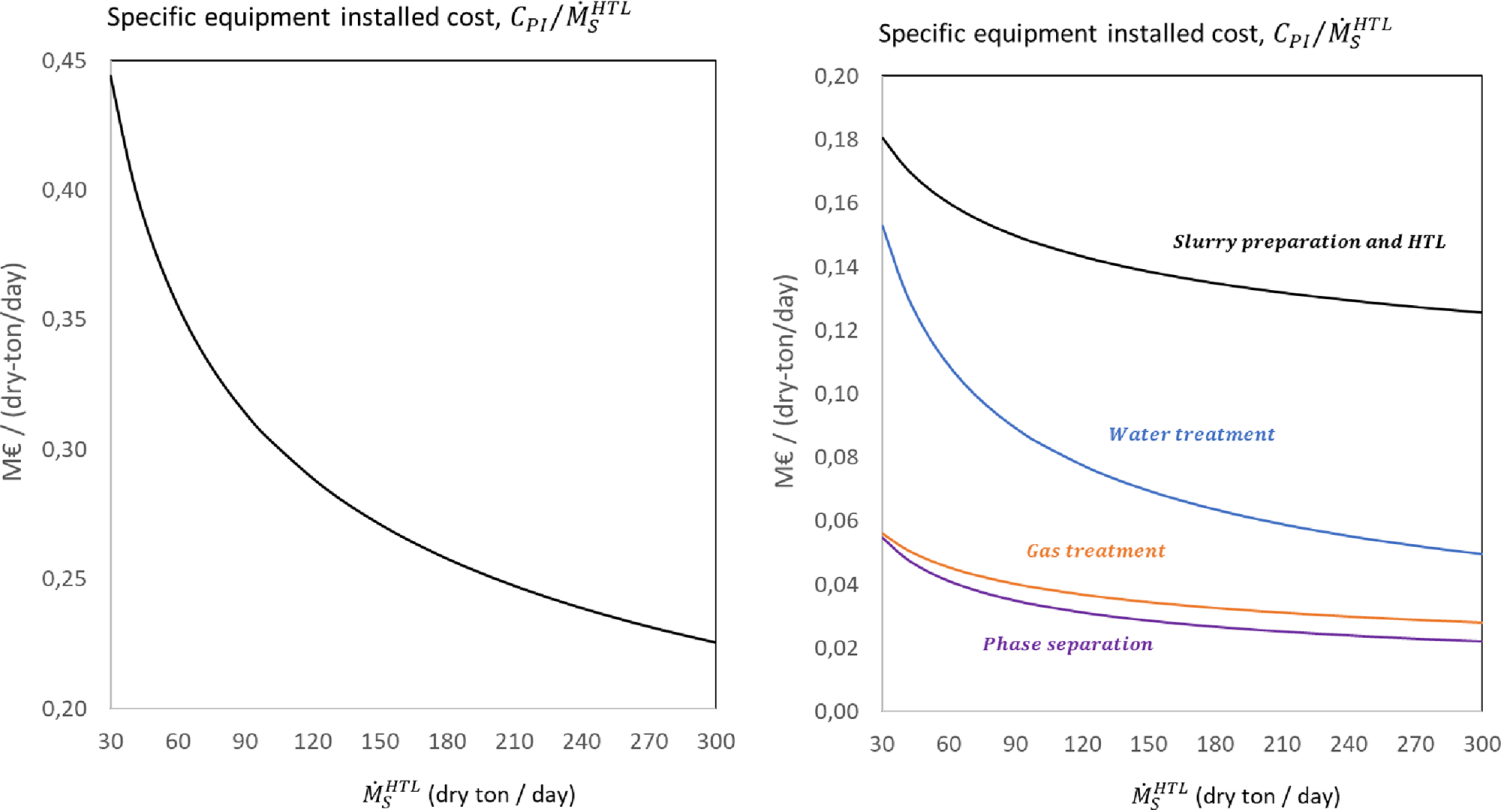
Variation of the specific installed equipment cost per unit mass flow rate of sewage sludge feed as a function of the biocrude production capacity: (left) total; (right) distribution among main systems, i.e., slurry preparation and HTL, phase separation, HTL water treatment, and HTL gas treatment.

**Figure 5:**
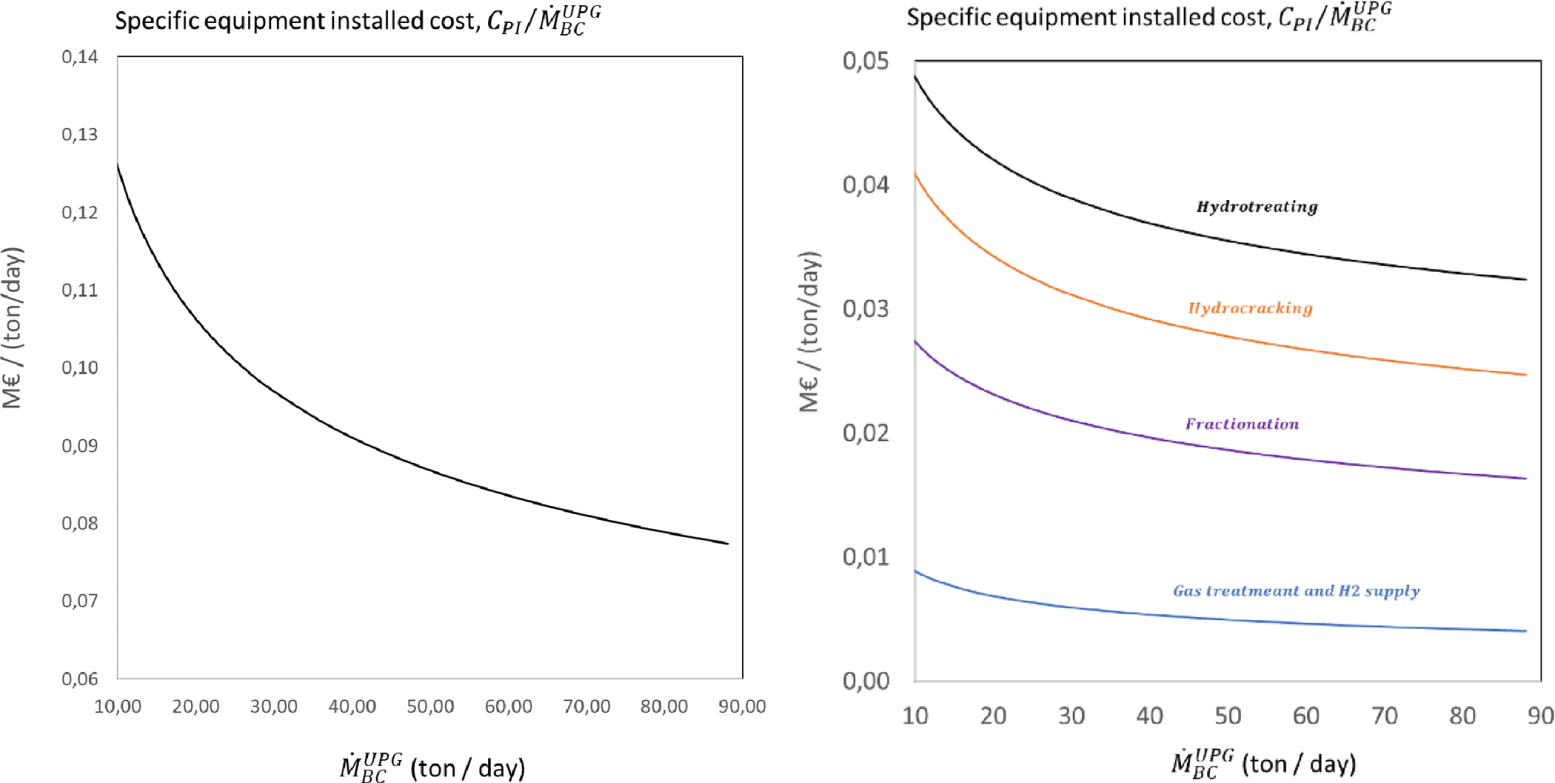
Variation of the specific installed equipment cost per unit mass flow rate of biocrude feed as a function of the biocrude upgrading capacity: (left) total; (right) distribution among main systems, i.e., hydrotreating (including the guard reactor), separation and fractionation, hydrocracking, and sour gas treatment with H2 recirculation.

Figure 4 shows the variation with the feed capacity of the total specific installed equipment cost per unit mass flow rate of dry feed of the complete biocrude production plant and for the main systems involved in the feedstock to biocrude conversion. The specific installed equipment cost for the biocrude production plant varies between 0.44 and 0.23 M€/dry-ton/day for plant capacities between 30 and 300 dry-ton/day. The largest contributions to the total installed equipment cost for the baseline design are the HTL and MVR units, representing approximately 70% of the total. As the plant capacity increases, the contribution from the MVR to the total installed cost also reduces due to its lower scale factor. The installed equipment cost for the overall biocrude production have a dependency with the plant capacity, which corresponds to an average scale factor of 0.7. Figure 5 shows the specific equipment installed cost per unit biocrude mass flow rate as a function of the feed capacity for the complete upgrading process and for the main systems. The total cost of equipment required for the upgrading varies between 130 and 80 k€/ton/day for the biocrude feed capacity range considered. The main contribution to the total equipment cost corresponds to the guard and hydrotreating system, representing approximately 44% of the total, followed by the hydrocracking and fractionation systems, which account for 25 and 26%. The cost of the equipment for cleaning and recycling the hydrogen-rich sour gas is relatively low, representing 5% of the total equipment cost. The installed equipment cost for the complete biocrude upgrading exhibits a power dependency with the feed capacity corresponding to an average scale factor of 0.78. The calculated values of the total permanent investment with the contribution of the different cost factors for the biocrude production and the biocrude upgrading are shown in Tables 15 and 16. All the costs associated to the development and construction of the biocrude production and the biocrude upgrading plants represent about 65% of the total permanent investment, approximately 80% of which is due to civil work, engineering, and contingencies.

**Table 15:** Annual Operating Cost and Income (M€/Year) for the Biocrude Production as a Function of the Feed Capacity

	30	50	100	150	200	250	300
total permanent investment	29.90	42.26	68.54	91.67	113.07	133.33	152.76
equipment installed cost	13.33	18.81	30.45	40.67	50.12	59.05	67.61
slurry preparation and HTL	5.41	8.25	14.72	20.76	26.55	32.17	37.66
phase separation	1.64	2.22	3.36	4.31	5.15	5.93	6.65
gas treatment	1.68	2.40	3.89	5.17	6.32	7.39	8.40
water treatment	4.59	5.95	8.48	10.43	12.09	13.56	14.89
chemicals (initial batch)	0.22	0.36	0.72	1.09	1.45	1.82	2.18
piping	0.87	1.22	1.98	2.64	3.26	3.84	4.39
electrical system	0.67	0.94	1.52	2.03	2.51	2.95	3.38
instrumentation & control system	0.60	0.85	1.37	1.83	2.26	2.66	3.04
project costs	14.22	20.08	32.50	43.40	53.48	63.01	72.15
land	1.33	1.88	3.05	4.07	5.01	5.91	6.76
site preparation	0.73	1.03	1.67	2.24	2.76	3.25	3.72
foundation and buildings	2.67	3.76	6.09	8.13	10.02	11.81	13.52
plant engineering	2.71	3.82	6.19	8.27	10.19	12.00	13.74
contingency	3.61	5.10	8.25	11.02	13.58	16.00	18.32
project development and licenses	0.73	1.03	1.67	2.23	2.75	3.24	3.71
commissioning	2.44	3.44	5.57	7.44	9.17	10.80	12.37
annual operating cost (M€)	2.56	3.85	6.89	9.81	12.67	15.48	18.27
consumables and utilities	0.867	1.445	2.895	4.350	5.809	7.271	8.738
base (NaOH) to HTL	0.044	0.073	0.146	0.219	0.292	0.365	0.438
catalyst (K_2_CO_3_) to HTL	0.087	0.146	0.291	0.437	0.583	0.728	0.874
acid to phase separation	0.032	0.054	0.108	0.162	0.216	0.270	0.324
MEK (phase separation)	0.000	0.000	0.000	0.000	0.000	0.000	0.000
natural gas	0.268	0.447	0.895	1.342	1.789	2.237	2.684
lime (gas cleaning)	0.005	0.008	0.016	0.024	0.033	0.041	0.049
NH_4_OH (25% NH_3_) to SCR	0.001	0.002	0.003	0.005	0.006	0.008	0.009
fresh water	0.000	0.000	0.000	0.000	0.000	0.000	0.000
electricity	0.429	0.715	1.435	2.160	2.890	3.623	4.359
labor	0.20	0.21	0.24	0.27	0.29	0.31	0.33
maintenance	0.27	0.38	0.61	0.81	1.00	1.18	1.35
insurance and taxes	0.60	0.85	1.37	1.83	2.26	2.67	3.06
administration and services	0.30	0.42	0.69	0.92	1.13	1.33	1.53
emissions to air	0.040	0.066	0.132	0.198	0.264	0.330	0.396
emissions to water	0.174	0.290	0.579	0.869	1.158	1.448	1.737
disposal of solid residue	0.113	0.188	0.377	0.565	0.753	0.942	1.130
income (M€)	3.12	5.20	10.40	15.60	20.80	26.00	31.20
sludge	3.12	5.20	10.40	15.60	20.80	26.00	31.20

**Table 16:** Annual Operating Cost and Income (M€/Year) as a Function of the Biocrude Feed Capacity

biocrude feed capacity (ton/day)	8.8	14.7	29.4	44	58.7	73.4	88.1
total permanent investment (M$)	2.60	3.43	5.85	8.05	10.11	12.08	13.98
equipment installed cost	1.16	1.71	2.91	3.99	5.01	5.98	6.93
hydrotreating	0.47	0.69	1.21	1.68	2.13	2.57	2.99
phase separation and fractionation	0.24	0.35	0.60	0.82	1.02	1.21	1.40
hydrocracking	0.37	0.54	0.92	1.26	1.58	1.89	2.18
sour gas treatment and hydrogen recycle	0.08	0.11	0.18	0.23	0.27	0.32	0.36
chemicals (initial batch)	0.02	0.03	0.06	0.08	0.11	0.14	0.17
piping	0.08	0.11	0.19	0.26	0.33	0.39	0.45
electrical system	0.06	0.09	0.15	0.20	0.25	0.30	0.35
instrumentation & control system	0.05	0.08	0.13	0.18	0.23	0.27	0.31
plant development costs	1.24	1.42	2.43	3.33	4.18	5.00	5.78
land	0.12	0.17	0.29	0.40	0.50	0.60	0.69
site preparation	0.06	0.09	0.16	0.22	0.28	0.33	0.38
foundation and buildings	0.23	0.34	0.58	0.80	1.00	1.20	1.39
plant engineering	0.24	0.26	0.44	0.60	0.75	0.90	1.04
contingency	0.31	0.34	0.58	0.80	1.00	1.20	1.39
project development and licenses	0.06	0.05	0.09	0.12	0.15	0.18	0.21
commissioning	0.21	0.17	0.29	0.40	0.50	0.60	0.69
total annual operating cost	4.48	5.84	8.42	10.43	12.13	13.63	14.97
consumables and utilities	4.150	5.432	7.814	9.635	11.153	12.469	13.639
biocrude	3.695	4.673	6.297	7.361	8.120	8.679	9.091
catalyst to guard reactor	0.029	0.048	0.096	0.144	0.192	0.240	0.288
catalyst to hydrotreating	0.023	0.039	0.078	0.117	0.156	0.195	0.235
catalyst to hydrocracking	0.011	0.019	0.037	0.056	0.074	0.093	0.112
make-up hydrogen	0.385	0.642	1.284	1.926	2.568	3.210	3.851
amine	0.003	0.005	0.011	0.016	0.022	0.027	0.032
fresh water	0.000	0.000	0.000	0.000	0.000	0.000	0.000
electricity	0.003	0.005	0.010	0.015	0.020	0.025	0.030
labor	0.17	0.17	0.17	0.17	0.17	0.17	0.17
maintenance	0.02	0.03	0.06	0.08	0.10	0.12	0.14
insurance and taxes	0.05	0.07	0.12	0.16	0.20	0.24	0.28
administration and services	0.03	0.03	0.06	0.08	0.10	0.12	0.14
emissions to air	0.058	0.097	0.194	0.290	0.387	0.484	0.581
emissions to water	0.003	0.004	0.009	0.013	0.017	0.021	0.026
income for selling of light gases	0.07	0.11	0.22	0.33	0.44	0.55	0.66

### 4.4 Operating Cost and Income

Tables 14 and 15 show the calculated results of the capital investment, annual operating cost, and annual income as a function of the feed capacity for the biocrude production and the biocrude upgrading, respectively. The annual operating costs are calculated as the sum of variable direct operational cost *C*_*op*, *d*_ dependent on the annual processing of feedstock, the fixed indirect operational costs *C*_*op*, *i*_ required for having the plant in activity, and the maintenance costs *C_maint_*. The direct operational costs include the purchase of consumables and utilities and the cost of the emissions to air and the disposal of solid residues and effluents. For the guard, hydrotreating, and hydrocracking reactors, the annual consumption of catalyst per unit hourly flow rate of the feed to the reactor is calculated in terms of the weight hourly space velocity of the catalyst from *Ṁ*_*cat*_^*S*^= (1/*WHSV*_*cat*_^*S*^)(*t_p_*/*t*_*cat*_^*S*^) with *t_p_* and *t*_*cat*_^*S*^denoting the annual production time and the life time of the catalyst (h). These costs are calculated based on the individual rates of consumption or production obtained from the mass and energy flows reported in Tables 8 and 9 with unit prices listed in Table 12 assuming an annual operating time of 8000 h. The annual indirect operational costs labor, administration, and insurance. The total annual labor cost has been evaluated from *C_labor_* = ∑*_j_N_j_*[*c*_*r*, *j*_(1 + *f_lb_*) + *f*_*OH*, *j*_*c_OH_*] , where the subscript *j* denotes the personnel categories, and *N_j_*, *c*_*r*, *j*_, *f_lb_*, *f*_*OH*, *j*_, and *c_OH_* represent the annual man-hour of personnel required, the hourly rate, the labor burden factor, the overhead factor, and the overhead cost factor, respectively. The personnel categories and the values for the labor cost used are shown in Table 13. The number of personnel required has been estimated based on individual main systems proportional to the purchase and installation costs, except for management, which is assumed to be constant. The costs for administration and insurance are evaluated as a percentage of the total permanent investment according to Table 13. The specific operating list for the biocrude production plant varies between 0.23 and 0.17 k€/dry-ton for feed capacities of sewage sludge in the range 30 to 300 dry-ton/day. Here, the cost of consumables and utilities represents approximately 25% of the total operating cost, with the electricity and the natural gas consumption contributing by 49 and 31%, respectively. The annual income from treating sewage sludge, considering a typical gate fee of 250 €/ton, can cover the total annual operating cost. Considering the biocrude upgrading in a centralized refinery, the specific operating cost varies between 1.65 and 0.54 k€/ton for biocrude feed capacities in the range of 8.8 and 88 ton/day, where about 84% of the total is due to the cost of the biocrude feed. The second main contribution is the cost of producing the make-up hydrogen from natural gas at the refinery, which represents approximately 4%. The annual cost of catalyst replacement, assuming a unit price of 31.4 €/liter and an operational lifetime of two years, represents only 1.1% to the total operating cost.

### 4.5 Levelized Cost of Biocrude and Minimum Fuel Selling Price

**Figure 6:**
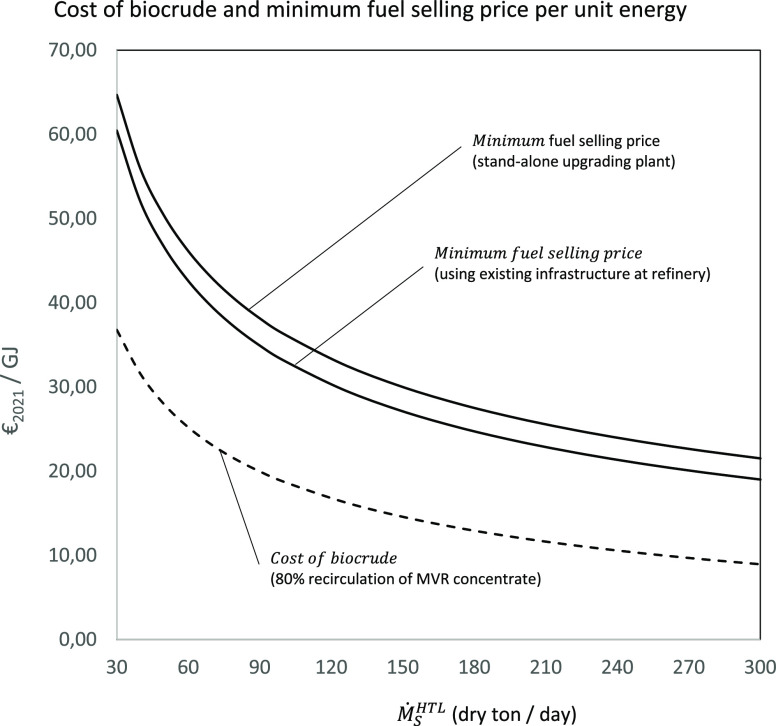
Variation as a function of the sewage sludge feed capacity of the levelized cost of biocrude production cost per liter of biocrude production (dashed line) and the minimum fuel selling price per liter (solid line) considering full investment in a new stand-alone upgrading unit and use of existing upgrading equipment at refinery.

The levelized costs of biocrude and the minimum fuel selling price (MFSP), denoted by *c_bc_* and *c_bf_*, are defined as the average prices for the biocrude and biofuels, respectively, per unit energy produced so that the overall net present value (NPV) for the total permanent investment and over its lifetime becomes zero. Based on these definitions, *c_bc_* and *c_bf_* are calculated using the formulas
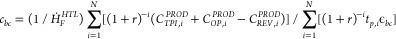
7and
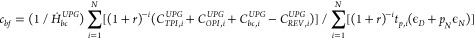
8Here, *r* is the expected return of investment, *C*_*TPI*, *i*_^*K*^, *C*_*OP*, *i*_^*K*^, and *C*_*REV*, *i*_^*K*^are the annual distributions of the annual total investment, operating costs, and revenues over the plant lifetime, with *K* = *PROD*, *UPG* denoting the biocrude production plant and the biocrude upgrading plant, respectively. In this notation, ϵ_*bc*_ is the annual energy efficiency of the sewage sludge to biocrude conversion, ϵ_*D*_ and ϵ_*N*_ are the annual energy efficiency of the biocrude to diesel and naphtha conversion, *p_N_* is the market price of naphtha relative to diesel, and *t*_*p*, *i*_ is the annual production time assumed to be 8000 h. The financial assumptions used in eqs 7 and 8 and the unit prices for calculation of revenues are shown in Table 14. Figure 6 shows the variation as a function of the sewage sludge feed capacity of the biocrude production cost and the minimum fuel selling price.

The results for the MFSP shown in Figure 6 have considered two different scenarios, i.e., full investment in a new stand-alone upgrading unit and use of existing upgrading equipment at refinery. In this second scenario, it is assumed that all equipment required for the upgrading of the biocrude are available at refinery and no capital investment is required, except for the initial batch of consumables, and only the annual operating costs and revenues are used for evaluating the minimum fuel selling price. The levelized cost of biocrude, which exhibits a monotonic decrease with the feed capacity, is in the range of 36.8 to 8.9 €_2021_/GJ per unit energy and 1.4 to 0.34 €_2021_/liter per unit volume for plant capacities between 30 and 300 dry-ton/day. These results are in agreement with the values found in the literature.^20,22^ If all the capital investment in a new upgrading plant is included, the average MFSP varies between 64.7 and 21.5 €_2021_/GJ per unit total energy of diesel and naphtha and 2.4 and 0.8 €_2021_/liter per unit volume of diesel equivalent for the range of the sewage sludge feed capacity used in the analysis. The parameters that impact the most on the MFSP are the biocrude price, the production cost of the make-up hydrogen, and the annual expenditure due to capital investment. Assuming that all equipment needed for upgrading of the HTL biocrude is available and can be utilized at the refinery with full replacement of fossil-derived feeds with HTL biocrude, the MFSP can be reduced only by approximately 6–7% for the production capacities considered. The results for the MFSP obtained from this analysis are also in line with the latest values obtained by Snowden-Swan et al.^20^

## 5 Conclusions

Production of liquid biofuels for road transportation can be achieved by (decentralized) direct conversion of the sewage sludge to an intermediate oil phase, so-called biocrude, via hydrothermal liquefaction at near-critical water conditions and further upgrading of the biocrude to naphtha and middle distillate at a centralized conventional refinery. The gas product from liquefaction can be co-combusted with natural gas for production of the net heat demand by the overall biocrude production process, which represents approximately 12% of the chemical energy contained in the sewage sludge. The aqueous effluent from liquefaction can be treated by air stripping for separation of the dissolved ammonia, which is combusted with the HTL gas, followed by mechanical vapor recompression. The overall mass and energy yields of biocrude are approximately 29.4 and 73.4% of the sewage sludge, respectively, with MBSP varying between 36.8 and 8.9 €_2021_/GJ per unit energy and between 1.4 and 0.34 €_2021_/liter per unit volume for sewage sludge feed capacities in the range of 30–300 dry-tons/day. The main costs contributing to the MBSP are the purchase and installation of the HTL process water treatment systems and capital cost associated to engineering and construction of the biocrude production plant. The overall upgrading process includes multi-stage catalytic hydrotreating of the biocrude for reduction of inorganics and S, N, and O heteroatoms, separation of the sour gas and water from the liquid oil, fractionation of the hydrotreated oil by distillation, and catalytic hydrocracking of the heavy distillate separated from fractionation. The main distillation products are naphtha and middle distillate, which represent gasoline and diesel pools, respectively, at the refinery. The overall mass and energy yields of combined naphtha and middle distillate from sewage sludge on dry basis is approximately 19 and 60%, where the naphtha fraction represents about 45% of the total. When considering investment in a new stand-alone unit for upgrading the biocrude, the minimum fuel selling price that can be achieved varies between 64.7 and 21.5 €_2021_/GJ per unit total energy of diesel and naphtha and 2.4 and 0.8 €_2021_/liter per unit volume of diesel equivalent for biocrude feed capacities in the range of 8.8 to 88 ton/day. The main contribution to the overall MFSP comes from the cost of biocrude, which represents about 60%, followed by the purchase and installation cost of process equipment. If existing equipment at refinery can be used for upgrading the biocrude, thus avoiding capital cost due to equipment and plant development and construction, the minimum fuel selling price reduces by 7%. Sewage sludge is considered a model urban waste feedstock posing the main challenges for the conversion to biofuels, i.e., high nitrogen and metal contents. Therefore, the overall production costs reported in this document are expected to be higher than those for biofuels produced from biocrude derived from other urbane waste fractions with lower contents of metals, N, S, and O.

## References

[ref1] A Roadmap for moving to a competitive low carbon economy in 2050; European Commission: Brussels, 2011; http://eur-lex.europa.eu/LexUriServ/LexUriServ.do?uri=COM:2011:0112:FIN:en:PDF

[ref2] De Jong S.; Hoefnagels R.; Faaij A.; Slade R.; Mawhood R.; Junginger M. The feasibility of short-term production strategies for renewable jet fuels – a comprehensive techno-economic comparison. Biofuels, Bioprod. Biorefin. 2015, 9, 778–800. 10.1002/bbb.1613.

[ref3] del Alamo G.; Kempegowda R. S.; Skreiberg Ø.; Khalil R. Decentralized Production of Fischer–Tropsch Biocrude via Co-processing of Woody Biomass and Wet Organic Waste in Entrained Flow Gasification: Techno-Economic Analysis. Energy Fuels 2017, 31, 6089–6108. 10.1021/acs.energyfuels.7b00273.

[ref4] Eurostat. Sewage Sludge Production and Disposal in the EU. 2020. Available online: http://appsso.eurostat.ec.europa.eu/nui/submitViewTableAction.do (accessed on 8 August 2020).

[ref5] Mantovi P.; Baldoni G.; Toderi G. Reuse of liquid, dewatered, and composted sewage sludge on agricultural land: effects of long-term application on soil and crop. Water Res. 2005, 39, 289–296. 10.1016/j.watres.2004.10.003. 15644237

[ref6] Singh R. P.; Agrawal M. Potential benefits and risks of land application of sewage sludge. Waste Manage. 2008, 28, 347–358. 10.1016/j.wasman.2006.12.010. 17320368

[ref7] Nielsen H. B.; Thygesen A.; Thomsen A. B.; Schmidt J. E. Anaerobic digestion of waste activated sludge—Comparison of thermal pretreatments with thermal inter-stage treatments. J. Chem. Technol. Biotechnol. 2011, 86, 238–245. 10.1002/jctb.2509.

[ref8] Fytili D.; Zabaniotou A. Utilization of sewage sludge in EU application of old and new methods—A review. Renewable Sustainable Energy Rev. 2008, 12, 116–140. 10.1016/j.rser.2006.05.014.

[ref9] Kelessidis A.; Stasinakis A. S. Comparative study of the methods used for treatment and final disposal of sewage sludge in European countries. Waste Manage. 2012, 32, 1186–1195. 10.1016/j.wasman.2012.01.012. 22336390

[ref10] Oladejo J.; Shi K.; Luo X.; Yang G.; Tao W. A Review of Sludge-to-Energy Recovery Methods. Energies 2019, 12, 60 10.3390/en12010060.

[ref11] Li Y.; Wang H.; Zhang J.; Wang J.; Ouyang L. The industrial practice of co-processing sewage sludge in cement kiln. Procedia Environmental Sciences 2012, 16, 628–632. 10.1016/j.proenv.2012.10.086.

[ref12] Park J. C.; Namkung H.; Yoon S.-P.; Seo H. S.; Xu L.-H.; Kim H.-T. Influence of phosphorus on ash fouling deposition of hydrothermal carbonization sewage sludge fuel via drop tube furnace combustion experiments. J. Energy Inst. 2020, 93, 2399–2408. 10.1016/j.joei.2020.07.014.

[ref13] Demirbas A.; Edris G.; Alalayah W. M. Sludge production from municipal wastewater treatment in sewage treatment plant. Energy Sources, Part A 2017, 39, 999–1006. 10.1080/15567036.2017.1283551.

[ref14] Sandquist J.; Tschentscher R.; del Alamo Serrano G. Hydrothermal liquefaction of organic resources in biotechnology: how does it work and what can be achieved?. Appl. Microbiol. Biotechnol. 2019, 103, 673–684. 10.1007/s00253-018-9507-2. 30474725

[ref15] Demirbas A. Thermochemical conversion of biomass to liquid products in the aqueous medium. Energy Sources 2006, 27, 1235–1243. 10.1080/009083190519357.

[ref16] Gollakota A. R. K.; Kishore N.; Gu S. A review on hydrothermal liquefaction of biomass. Renewable Sustainable Energy Rev. 2018, 81, 1378–1392. 10.1016/j.rser.2017.05.178.

[ref17] Jensen C. U.; Guerrero J. K. R.; Karatzos S.; Olofsson G.; Iversen S. B. Fundamentals of Hydrofaction^TM^: Renewable crude oil from woody biomass. Biomass Convers. Biorefin. 2017, 7, 495–509. 10.1007/s13399-017-0248-8.

[ref18] Kruse A.; Dinjus E. Hot compressed water as reaction medium and reactant 2. Degradation reactions. J. Supercrit. Fluids 2007, 41, 361–379. 10.1016/j.supflu.2006.12.006.

[ref19] Snowden-Swan L. J.; Zhu Y.; Jones S. B.; Elliott D. C.; Schmidt A. J.; Hallen R. T.; Billing J. M.; Hart T. R.; Fox S. P.; Maupin G. D. Hydrothermal Liquefaction and Upgrading of Municipal Wastewater Treatment Plant Sludge: A Preliminary Techno-Economic Analysis, Rev.1; Pacific Northwest National Lab: United States: N. p., 2016. Web. 10.2172/1327165.

[ref20] Snowden-Swan L. J.; Shuyun L.; Jiang Y.; Thorson M; Schmidt A; Seiple T.; Billing J.; Santosa M; Hart T.; Fox S.; Cronin D.; Ramasamy K.; Anderson D.; Hallen R.; Fonoll Almansa X.; Norton J. Wet waste hydrothermal liquefaction and biocrude upgrading to hydrocarbon fuels: 2021 state of technology; Pacific Northwest National Lab 2022. 10.2172/1863608

[ref21] Jones S.; Zhu Y.; Anderson D.; Hallen R.; Elliott D.; Schmidt A.; Albrecht K.; Hart T.; Butcher M.; Drennan C.; Snowden-Swan L.; Davis R.; Kinchin C. Process Design and Economics for the Conversion of Algal Biomass to Hydrocarbons: Whole Algae Hydrothermal Liquefaction and Upgrading; Pacific Northwest National Laboratory: Richland, WA. 2014 PNNL-23227.

[ref22] Li S.; Jiang Y.; Snowden-Swan L. J.; Askander J. A.; Schmidt A. J.; Billing J. M. Techno-economic uncertainty analysis of wet waste-to-biocrude via hydrothermal liquefaction. Appl. Energy 2021, 283, 11634–11655.

[ref23] Thomas D. C. Transport Characteristics of Suspensions, VIII, A Note on the Viscosity of Newtonian Suspensions of Uniform Spherical Particles. J. Colloids Sci. 1965, 20, 267–277. 10.1016/0095-8522(65)90016-4.

[ref24] International Association for the Properties of Water and Steam, IAPWS R16–17(2018), Revised Release on the IAPWS Formulation 2017 for the Thermodynamic Properties of Heavy Water; IAPWS (2018)

[ref25] NIST database of thermophysical Properties of Fluid Systems. https://webbook.nist.gov/chemistry/fluid/

[ref26] Sayegh A.; Prakash N. S.; Pedersen T. H.; Horn H.; Saravia F. Treatment of hydrothermal liquefaction wastewater with ultrafiltration and air stripping for oil and particle removal and ammonia recovery. J. Process Eng. 2021, 44, 102427 10.1016/j.jwpe.2021.102427.

[ref27] Schwantes R.; Chavan K.; Winter D.; Felsmann C.; Pfafferott J. Techno-economic comparison of membrane distillation and MVC in a zero liquid discharge application. Desalination 2018, 428, 50–68. 10.1016/j.desal.2017.11.026.

[ref28] Ito S.; Uchida M.; Onishi S.; Kato S.; Toshiro F.; Kobayashi Hideaki, ″Performance of Ammonia-Natural Gas Co-Fired Gas Turbine for Power Generation,″ 15th Annual NH3 Fuel Conference, Pittsburgh, PA; IHI Corporation: October 31, 2018.

[ref29] Rosenberg H. S.; Oxley J. H.. Selective Catalytic Reduction for NOx Control at Coalfired Power Plants. ICAC Forum ‘93, Controlling Air Toxics and NOx Emissions; Baltimore, MD, February 24–26, 1993

[ref30] Dutta A.; Sahirm A.; Tan E.; Humbird D.; Snowden-Swan L. J.; Meyer P. A.; Ross J.; Sexton D.; Yap R.; Lukas J. Process design and economics for the conversion of lignocellulosic biomass to hydrocarbon fuels: Thermochemical research pathways with in-situ and ex-situ upgrading of fast pyrolysis vapors; Pacific Northwest National Lab. (PNNL): Richland, WA, 2015

[ref31] Jones D. A. ″Technoeconomic Evaluation of MEA versus Mixed Amines and a Catalyst System for CO2 Removal at Near-Commercial Scale at Duke Energy Gibson 3 Plant and Duke Energy Buck NGCC Plant″ Lawrence Livermore National Laboratory Report LLNL-TR-758732. 2018

[ref32] Colebrook C. F.; White C. M. Experiments with Fluid Friction in Roughened Pipes. Proc. R. Soc. London, Ser. A 1937, 161, 367–381.

[ref33] Peters M. S.; Timmerhaus K. D.; West R. E. Plant design and economics for chemical engineers; 5th ed.; McGraw-Hill: New York, 2003; vol. 4.

[ref34] Woods D. R. Rules of Thumb. Rules of Thumb in Engineering Practice; Wiley-VCH: 2008

[ref35] Kazi F. K.; Fortman J.; Anex R.; Kothandaraman G.; Hsu D.; Aden A.; Dutta A. ″Techno-Economic Analysis of Biochemical Scenarios for Production of Cellulosic Ethanol,″ Technical Report NREL/TP-6A2–46588, June 2010. https://www.nrel.gov/docs/fy10osti/46588.pdf

[ref36] Knorr D.; Lukas J.; Schoen P. Production of Advanced Biofuels via Liquefaction. Hydrothermal Liquefaction Reactor Design. NREL/SR-5100-60462, Nov. 2013. https://www.nrel.gov/docs/fy14osti/60462.pdf

[ref37] Stubenvoll J.; Böhmer S.; Szednyj I. ″State of the Art for Waste Incineration Plants″ Federal Ministry of Agriculture and Forestry, Environment and Water Management: Vienna 2002. ISBN 3–902 338–13-X

